# Effect of *Bojanggunbi-tang* and its primary constituent herbs on the gastrointestinal tract: a scoping review

**DOI:** 10.3389/fphar.2025.1543194

**Published:** 2025-03-12

**Authors:** Chaehyun Park, Minjeong Kim, Jae-Woo Park, Jinsung Kim, Youngmin Bu, Seok-Jae Ko

**Affiliations:** ^1^ Department of Clinical Korean Medicine, Graduate School, Kyung Hee University, Seoul, Republic of Korea; ^2^ Department of Korean Internal Medicine, Kyung Hee University College of Korean Medicine, Kyung Hee University Hospital at Gangdong, Seoul, Republic of Korea; ^3^ Department of Digestive Diseases, College of Korean Medicine, Kyung Hee University, Seoul, Republic of Korea; ^4^ Division of Digestive Diseases, Department of Korean Internal Medicine, Kyung Hee University Korean Medicine Hospital, Seoul, Republic of Korea; ^5^ Department of Herbal Pharmacology, College of Korean Medicine, Kyung Hee University, Seoul, Republic of Korea

**Keywords:** *Bojanggunbi-tang*, herbal medicine, gastrointestinal, *Lonicera japonica*, *Atractylodes macrocephala*, *Alisma canaliculatum*

## Abstract

**Background:**

*Bojanggunbi-tang* (BGT), a herbal prescription used in traditional Korean medicine, has been used to treat various gastrointestinal (GI) diseases.

**Methods:**

Studies on BGT published until May 2024 were retrieved from the electronic databases of Medline, CENTRAL, Embase, AMED, CNKI, CiNii, Kmbase, KISS, NDSL, and OASIS using GI-related terms. All study types, regardless of the research method or language, were eligible for inclusion. Additional articles on *Lonicera japonica*, *Atractylodes macrocephala*, and *Alisma canaliculatum*, which are key components of BGT, were retrieved from the databases of Medline, CENTRAL, Embase, and Web of Science using GI-specific terms. The basic information, research models, administration methods, evaluation methods, and treatment outcomes of the selected studies were examined subsequently.

**Results:**

Fourteen studies, comprising nine animal studies, one cell-based study, and four human studies, were included in the final analysis. BGT was found to exhibit anti-inflammatory effects, promote restoration of the gastrointestinal mucosa, and regulate GI motility. Analysis of the key herbal components *L. japonica*, *A. macrocephala*, and *A. canaliculatum* revealed that they inhibit inflammatory cytokines and oxidative substances, regulate serotonin and cholinergic pathways, and modulate intestinal microbiota.

**Conclusion:**

This scoping review confirmed the therapeutic potential and mechanisms of action of BGT and its main components, *L. japonica*, *A. macrocephala*, and *A. canaliculatum,* thereby indicating its ability to enhance GI health. Further studies, including randomized clinical trials, must be conducted in the future to confirm these findings.

**Scoping review registration:**

The study was registered in OSF, an international scoping review database: https://doi.org/10.17605/OSF.IO/ATU4S.

## 1 Introduction

*Bojanggunbi-tang* (BGT; 補腸健脾湯) is a combination of the herbal prescriptions “*Daehwajungeum*” and “*Sambaek-tang*” from ancient Chinese medical literature. It comprises 16 herbs, namely, *Lonicera japonica, Atractylodes macrocephala, Paeonia lactiflora, D. lablab, Dioscorea japonica, Crataegus pinnatifida, Poria cocos, Magnolia officinalis, Citrus unshiu, Alisma canaliculatum, Massa medicata, Hordeum vulgare, Zingiber officinale, Aucklandia lappa, Amomum villosum,* and *Glycyrrhiza uralensis*. BGT has been used to treat several diseases, such as acute gastritis, colitis, inflammatory bowel disease (IBD), and gastrointestinal (GI) symptoms (e.g., abdominal pain, indigestion, and diarrhea) in Korea (Joun et al., 1994). Numerous herbs have been used to tonify the spleen and replenish qi (補脾益氣), tonify blood and yin (補血養陰), and induce diuresis to drain dampness (). BGT relieves dampness and improves deficiency syndromes such as spleen deficiency and yin or yang deficiency patterns (Nam et al., 1989). Ryu et al. revealed that BGT was prescribed to over 310,000 patients with IBD or other forms of colitis at a Korean Medical Center between 1994 and 2010 (Ryu et al., 2011). A modified version of BGT (mBGT), comprising the herbs *L. japonica, A. macrocephala,* and *A. canaliculatum*, has demonstrated equally favorable effects on colitis (Kim et al., 2021). However, no randomized controlled trial (RCT) or systematic review (SR) has evaluated the efficacy of this formulation, thereby limiting the comprehensive understanding of its effects and mechanisms.

Scoping reviews constitute a flexible and rigorous approach to synthesizing evidence that can address broader research questions (Rodger et al., 2024). Scoping reviews identify different types of evidence available in a particular field, summarize existing evidence, and make recommendations for future research (Tricco et al., 2016). This scoping review summarized the findings of clinical and basic studies on BGT and its main herbal components, *L. japonica, A. macrocephala,* and *A. canaliculatum,* which have demonstrated efficacy in previous experimental studies. Furthermore, the present study also analyzed the characteristics and mechanisms of BGT and its constituent herbs as treatments for GI dysfunction to clarify their efficacy and provide insights for future research.

## 2 Methods

This review adhered to the Preferred Reporting Items for Systematic Reviews and Meta-Analysis (PRISMA) Extension for Scoping Reviews (PRISMA-ScR), a reporting guideline for scoping reviews (Supplementary Table S1). It was also registered in OSF, an international scoping review database (https://doi.org/10.17605/OSF.IO/ATU4S).

Articles related to BGT published up to 2024 were retrieved from the databases of MEDLINE (via PubMed), Cochrane Central Register of Controlled Trials (CENTRAL), EMBASE, Allied and Complementary Medicine Database (AMED), China National Knowledge Infrastructure (CNKI), Citation Information by Nii (CiNii), Korean Medical Database (Kmbase), Korean Studies Information Service System (KISS), National Digital Science Library (NDSL), and Oriental Medicine Advanced Searching Integrated System (OASIS). No restrictions were applied in terms of language or publication dates.

**FIGURE 1:**
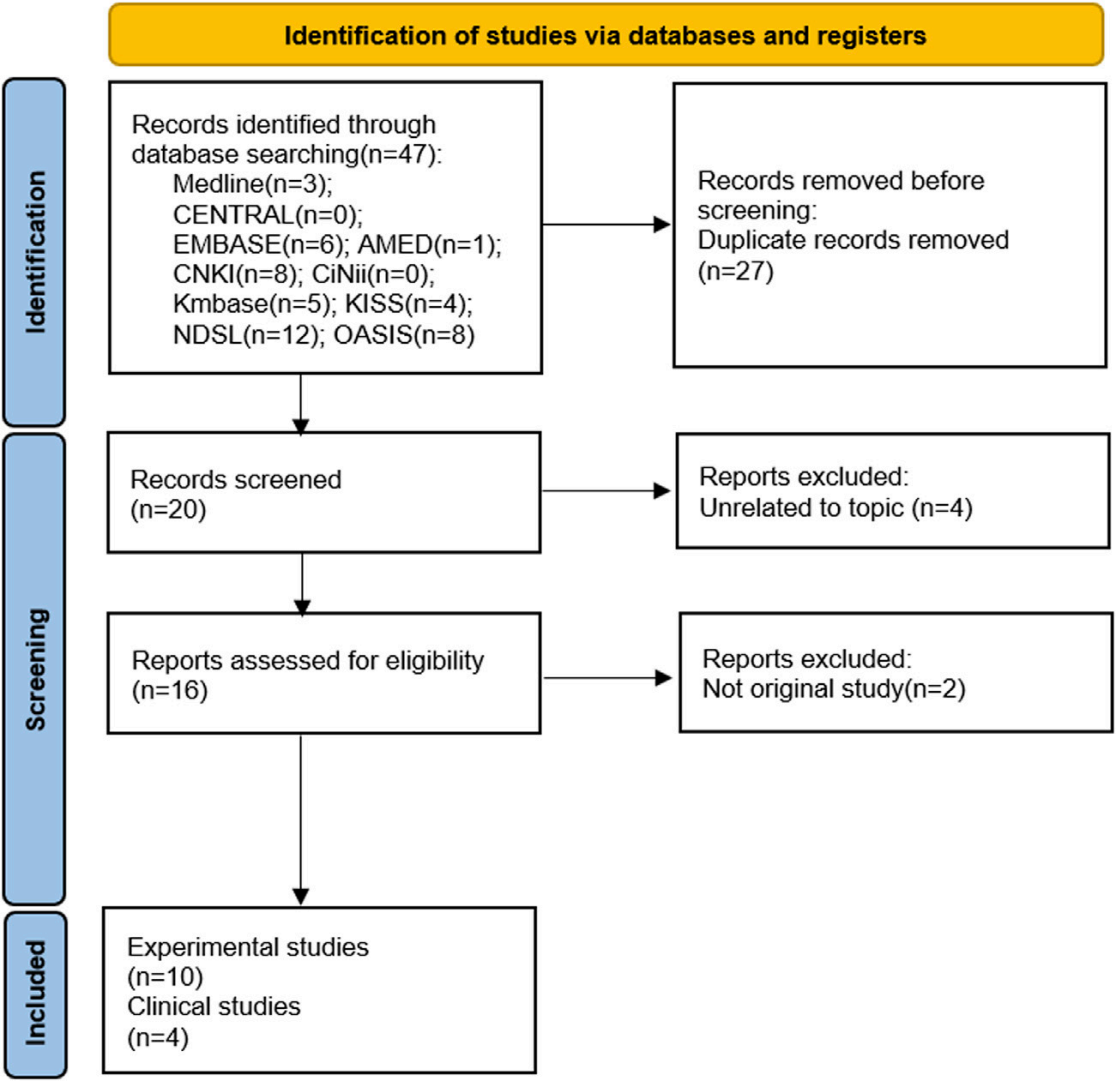
Preferred Reporting Items for Systematic reviews and Meta-Analyses (PRISMA) flow checklist for the study.

The search terms used to retrieve the articles were as follows: “Bojanggunbi-tang,” “Bojanggunbitang,” “Bojangkunbi-tang,” and “補腸健脾湯.” In addition, the following terms related to GI conditions were also used to retrieve articles: “gastrointestinal,” “gastr*,” “intestin*,” “colon,” “bowel,” “colitis,” and “Crohn” (Supplementary Table S2). All types of studies, including *in vitro*, *in vivo*, and clinical studies, that focused on BGT were eligible for inclusion. Fourteen studies, comprising one *in vitro* study, nine animal studies, and four human studies (Figure 1), were retained after removing duplicates and unrelated studies.

Three key herbal components of BGT, namely, *L. japonica Thunb.* (LJT) and *A. macrocephala Koidz.* (AMK), and *A. canaliculatum A. Braun & C.D.Bouche* (AC), were identified through further analysis. An additional search was conducted to retrieve articles related to these herbs from the databases of MEDLINE (via PubMed), CENTRAL, EMBASE, and the Web of Science published until May 2024 to better understand their individual roles. The search terms used to retrieve the articles were as follows: “*L. japonica*,” “Atractylodes macrocephala,” and “Alisma,” and GI-related terms such as “gastrointestinal,” “gastr*,” “intestin*,” “colon,” “bowel,” “colitis,” and “Crohn” (Supplementary Figure S2). Studies unrelated to GI function or studies evaluating these herbs in combination with other herbs were excluded. This secondary search yielded 11 studies on LJT, 37 studies on AMK, and two studies on AC, which were included in the final analysis (Supplementary Figures S1–S3).

The basic information, research models, administration methods, evaluation methods, and treatment outcomes of each of the selected studies were evaluated. Basic study information included the year of publication and study design. The methods of administering the herbal extracts were analyzed according to the extraction method, concentration, dosage, route of administration, duration, and number of sessions.

## 3 Results

### 3.1 Preclinical studies

Several studies have assessed the efficacy of BGT and its modified forms (Table 1). The first study exploring the effects of BGT on the GI tract was conducted by Nam et al. (1989). They measured the levels of gastric juice secretion, acidity, the area of ethanol-induced ulcers, intestinal transportability, and anti-cathartic action to evaluate the effects of BGT. BGT exerted a significant inhibitory effect on gastric juice secretion, reduced acidity, and alleviated ethanol-induced gastric ulcers. Similarly, the study conducted by Joun et al. (1994) revealed that BGT prevented the formation of ulcers induced by pylorus ligation and indomethacin. Furthermore, it exerted inhibitory effects on gastric juice secretion and smooth muscle contractions in the ileum and stomach, reduced free and total acidity, and decreased the transport ability in the large intestine. Ro et al. (2006) demonstrated that BGT inhibited smooth muscle contractions in the ileum, indicating its anti-cathartic effects. They further reported that BGT suppressed transportability in the small and large intestines and prevented diarrhea induced by castor oil, pilocarpine, and barium chloride. Kim et al. (1993) investigated the effects of several traditional herbal medicines used as energy invigorators, including BGT, on serum components and intestinal bacteria. BGT increased the count of lactic acid bacteria in the intestinal flora; furthermore, it regulated the fecal β-glucuronidase activity. Collectively, the findings of these studies indicate that BGT exerts an anti-ulcer effect in the stomach by suppressing the secretion of gastric juice. BGT exhibits anti-diarrheal effects by regulating intestinal transportability and fostering a balance in the intestinal microenvironment. However, studies conducted before 2000 reported conflicting results regarding the transportability and anti-diarrheal effects of BGT, likely due to biases introduced by the experimental methods used. For transportability assessment, the distance traveled by orally administered solutions was calculated through GI tract resection, a method that does not accurately reflect the transportability of each organ. Similarly, the anti-diarrheal effects were subjectively scored based on stool form, leading to a lack of objectivity and reproducibility. In contrast, studies conducted since the 2000s have shown more consistent results by employing more robust and objective measures, such as inflammation markers and histological scores.

**TABLE 1:** Preclinical studies on *Bojanggunbi-tang*.

Study	Model	Species or cell	Inducer	Dose/Route/Regimen	Results
*in vivo*					
Nam et al. (1989)	—	SD rats (200–220 g)	—	BGT water extract (454.4 mg/200 g) directly injected into the duodenum 7 h before testing	BGT • Inhibited gastric juice secretion • Lowered gastric juice acidity
	Ethanol ulcer	SD rats (200–220 g)	Oral administration of 1 mL 99.5% ethanol	BGT water extract (454.4 mg/200 g) p.o. 1.5 h before testing	BGT prevented ulcer formation
	—	ICR mice (20–22 g)	Oral administration of 10% charcoal liquid suspended in 0.5% methylcellulose	BGT water extract (45.4 mg/20 g) p.o. 50 min before testing	BGT did not activate transportability of the small intestine
	Diarrhea	ICR mice (20–22 g)	Oral administration of 0.2 mL/20 g castor oil	BGT water extract (45.4 mg/20 g) p.o. 30 min before testing	BGT did not exhibit anti-cathartic effect
Kim et al. (1993)	—	ICR mice (18–25 g)	—	BGT water extract (2 g/kg) for 14 days before testing	BGT • Inhibited feces β-glucuronidase activation • Increased the count of lactic acid bacteria in the intestinal flora
	Antibiotics-pretreatment	ICR mice (18–25 g)	Oral administration of chloramphenicol (17.5 mg), nystatin (500 IU), streptomycin (20 mg), erythromycin (10 mg), penicillin G (200 IU) mix 0.1 mL/10 g for 3 days	BGT water extract (2 g/kg) for 7 days before testing	BGT increased feces β-glucuronidase activity
Joun et al. (1994)	Ileum and stomach smooth muscle contraction	Ileum and anterior stomach tissue of Male ICR mice (18–24 g)	Acetylcholine chloride and Barium chloride	BGT water extract	BGT • Inhibited ileum smooth muscle contraction • Inhibited stomach contraction
	Pylorus ligation ulcer	Male SD rats (180–220 g)	Pylorus ligation	BGT water extract (900 mg and 1,800 mg/kg) i.p. immediately before testing	BGT prevented ulcer formation
	Indomethacin-induced ulcer	Male SD rats (180–220 g)	Indomethacin (25 mg/kg) injected subcutaneously	BGT water extract (900 mg and 1,800 mg/kg) p.o. 1 h before testing	BGT prevented ulcer formation
	—	Male ICR mice (18–24 g)	—	BGT water extract (900 mg and 1,800 mg/kg) i.p. immediately before testing	BGT • Inhibited gastric juice secretion • Increased pepsin output
		Small intestine of Male ICR mice (18–24 g)	Oral administration of 25% barium sulfate (0.2 mL/mouse)	BGT water extract (900 mg and 180 0 mg/kg) p.o. 30 min before challenge	BGT did not affect transportability of the small intestine
		Large intestine of Male ICR mice (18–24 g)	Oral administration of 25% barium sulfate (0.1 mL/10 g)	BGT water extract (900 mg and 1,800 mg/kg) p.o. 30 min before challenge	BGT activated transportability of the large intestine
Ro et al. (2006)	Ileum smooth muscle contraction	Ileal tissue of Male ICR mice (18–24 g)	Barium chloride and histamine	BGT water extract (200 mg, 600 mg/kg)	BGT inhibited ileum smooth muscle contraction
		Male guinea pig (250–300 g)			
	—	Male ICR mice (18–24 g)	Oral administration of 25% BaSO_4_ (0.2 mL/mouse)	BGT water extract 200 mg/kg and 600 mg/kg p.o. 30 min before challenge	BGT suppressed the small intestine transportability
	—	Male ICR mice (18–24 g)	Oral administration of 25% BaSO_4_ (0.2 mL/mouse) and 15 min after the subcutaneous injection of 50 μg/kg of neostigmine	BGT water extract 200 mg/kg and 600 mg/kg p.o. 30 min before challenge	BGT suppressed the small intestine transportability
	—	Male ICR mice (18–24 g)	Oral administration of 25% BaSO_4_ 0.1 mL/10 g	BGT water extract 200 mg/kg and 600 mg/kg p.o. 30 min before challenge	BGT suppressed the large intestine transportability
	Diarrhea	Male ICR mice (18–24 g)	Oral administration of45% castor oil 0.1 mL/10 g	BGT water extract 200 mg/kg and 600 mg/kg 3 h before challenge	BGT showed anti-cathartic effects
			Subcutaneous injection of pilocarpine 32 mg/kg		
			Subcutaneous injection of barium chloride 45 mg/kg		
Ryu et al. (2011)	Ulcerative colitis	Male ICR mice (21–25 g)	Oral administration of 5% DSS ad libitum for 7 days	BGT water extract 50, 150, and 450 mg/kg p.o. (two times every 7 days)	BGT • Inhibited weight loss and colon length shortening • Reduced histological damage of the colon tissue
	Crohn’s disease	Male ICR mice (21–25 g)	Intrarectally administrated 2.5% TNBS in 50% ethanol solution	BGT water extract 50, 150, and 450 mg/kg p.o. (two times every 3 days)	BGT • Inhibited colon length shortening • Improved survival rate
Ko et al. (2017)	Murine colitis	Balb/c mice	Oral administration of distilled water with DSS for 7 days	BGT-E 30, 100 mg/kg and 300 mg/kg p.o. for 7 days	BGT-E • Prevented colon length shortening and histological damage • Decreased the IL-1β, TNF-α and IL-17 levels
Kim (2017)	Colitis	Male BALB/c mice (20–24 g)	Oral administration of 5% DSS for 7 days	Eight major herbs of BGT water extract 300 mg/kg p.o. (two times every 7 days)	LJ extract and AO extract • Inhibited colon length shortening • Reduced colon tissue’s histological damage
				mBGT (1:1:1, 3:1:1) water extract 300 mg/kg p.o. (two times every 7 days)	mBGT inhibited colon length shortening mBGT (3:1:1) reduced histological damage of the colon tissue
				mBGT (3:1:1) water extract 30, 100, and 300 mg/kg p.o. (two times every 7 days)	mBGT • Inhibited colon length shortening • Decreased the IL-1β, TNF-α and IL-17 levels • Improved colorectal tissue and fecal condition • Reduced histological damage of the colon tissue
Kim et al. (2021)	Colitis	Male BALB/c mice (20–24 g)	Oral administration of 5% DSS ad libitum for 7 days	mBGT water extract 30, 100, and 300 mg/kg p.o. (two times every 7 days)	mBGT • Inhibited colon length shortening • Improved colorectal tissue and fecal condition • Reduced histological damage of the colon tissue • Decreased IL-1β, TNF-α and IL-17 levels
Cho et al. (2022)	NSI	C57B/L male mice	Subcutaneous injection of 15 mg/kg indomethacin	BGT water extract 50, 150, and 450 mg/kg p.o. 30 min before and 6 h after challenge	BGT • Inhibited colon length shortening • Reduced ulceration area • Improved histological damage related to inflammation and ulcer
*in vitro*					
Choi et al. (2023)	—	Small intestine interstitial cells of Cajal from ICR mice	—	BGT water extract 1, 5, and 10 mg/mL	BGT • Depolarized pacemaker potential • Reduced firing frequency BGT acts through the CHRM3, 5-HT3, and 5-HT4 receptors to regulate intracellular Ca2+ concentrations and PKC, MAPK, guanylate cycle, and PKG signaling pathways

SD, sprague dawley; ICR, institute of cancer research; p. o., per oral; i. p., intraperitoneal administration; BGT, Bojanggunbi-tang; mBGT, modified bojanggunbi-tang; BGT-E, Bojanggunbi-tang essence; min, minute; h, hour; LJ, *Lonicera japonica*; AO, *Alisma Orientalis*; DSS, dextran sulfate sodium; TNBS, 2,4,6-Trinitrobenzene sulfonic acid; CCl_4_, carbon tetrachloride; BaSO_4_, barium sulfate; NSI, Non-steroidal anti-inflammatory drug-induced small intestinal injury; CHRM3, Cholinergic receptor muscarinic 3; 5-HT, 5-hydroxytryptamine; PKC, Protein kinase C; MAPK, Mitogen-activated protein kinase; PKG, Protein kinase G.

Most subsequent studies focused on the efficacy of BGT in the treatment of colitis. Most of these studies employed colitis models created using dextran sulfate sodium (DSS), a substance that is toxic to the gut epithelium. DSS induced acute colitis in mice by compromising the mucosal barrier. Acute colitis in mice exhibits symptoms similar to those of human ulcerative colitis, such as weight loss, colon shortening, diarrhea, bloody stools, and colonic mucosal ulceration (Eggera et al., 2000).

Ryu et al. (2011) examined the effect of BGT on DSS-induced ulcerative colitis and a 2,4,6-trinitrobenzene sulfonic acid (TNBS)-induced CD model. The administration of BGT for 7 days following the induction of colitis revealed the protective effects of BGT against weight loss, colon shortening, and histological changes in the colon, as well as the inhibition of key inflammatory cytokines in the DSS model. BGT prevented colon shortening and improved the survival rate in the TNBS model. Significant improvements in colitis were also observed following the administration of BGT essence and modified BGT (mBGT) in the study by Ko et al., as evidenced by increased colon length, better pathology scores, reduced histological damage, and inhibition of pro-inflammatory cytokines in DSS models (Ko et al., 2017).

Studies on mBGT (Kim, 2017; Kim et al., 2021) have led to the identification of a more potent and cost-effective combination of BGT. The dried flower buds or flowers of *L. japonica Thunb.*, dried rhizome of *A. macrocephala Koidz.*, and dried rhizome of *Alisma orientalis Juz.* were prepared as aqueous extracts, and mixed at a ratio of 3:1:1 (the original BGT ratio) and 1:1:1 in a preliminary study conducted by Kim (Kim, 2017) to explore the effects of eight major herbs on BGT. Comparison of these ratios revealed that the 3:1:1 mixture was more effective in preventing colon shortening and improving histological changes. Moreover, the effects of the modified 3:1:1 ratio were superior to those of BGT in terms of reducing colon shortening and improving anatomical and histological scores. Kim et al. (2021) also compared BGT with mBGT at the 3:1:1 ratio and revealed that both formulations improved colon shortening, macroscopic scores, histological damage, clinical scores, and cytokine levels (including the interleukin (IL)-1β, tumor necrosis factor (TNF)-α and IL-17 levels). However, the performance of mBGT was superior to that of BGT, especially in terms of clinical and histological improvements. Thus, mBGT may be a more effective treatment option for patients with colitis.

Two recent studies examined the effects of BGT on the small intestine (Cho et al., 2022; Choi et al., 2023). Cho et al. (2022) used an indomethacin-induced murine colitis model to study the effects of BGT on non-steroidal anti-inflammatory drug (NSAID)-induced small intestinal injury (NSI). The administration of BGT before and after the induction of NSI yielded a reduction in small intestine shortening, ulceration area, and inflammation scores. Choi et al. (2023) explored the regulatory mechanisms of BGT on the interstitial cells of Cajal (ICCs) in the small intestine, the pacemaker cells of the GI tract. The ICC pacemaker potential measured using an electrophysiological method revealed that higher BGT concentrations induced depolarization and decreased the firing frequency in ICCs. Various receptor antagonists used to identify the mechanisms underlying the effects of BGT on ICCs have revealed that BGT acts through the cholinergic receptor muscarinic (CHRM) 3, 5-hydroxytryptamine (5-HT) 3 receptors, and 5-HT4 receptors. Thus, BGT regulates the intracellular Ca^2+^ levels and activates signaling pathways involving protein kinase C (PKC), mitogen-activated protein kinase (MAPK), the guanylate cycle, and protein kinase G (PKG) through these receptors. CHRM 3 induces smooth muscle contraction and gland secretion (Abrams et al., 2006). Serotonin (5-HT), a monoamine neurotransmitter, regulates intestinal motility. The 5-HT3 and 5-HT4 receptors are key mediators of peristaltic contractions in mammals (Sia et al., 2013). Thus, BGT depolarizes ICCs through the cholinergic and serotonergic pathways, thereby promoting GI movement.

In summary BGT and its modified forms exhibit significant anti-inflammatory and mucosal protective effects throughout the GI tract. Thus, BGT can be used to treat IBD, gastric ulcers, and small intestinal injuries.

### 3.2 Clinical studies

Four clinical studies, including two case reports, were reviewed in the present study (Table 2). Two retrospective chart reviews analyzed the effectiveness of various herbal medicines, including BGT, in the treatment of irritable bowel syndrome (IBS).

**TABLE 2:** Clinical studies on *Bojanggunbi-tang*.

Study	Study Type	Sample	Treatment	Duration of Administration	Evaluation	Result
Chang et al. (1985)	RCR	116 patients with IBS	Eight prescriptions including BGT	Mostly 10–19 days	Total effective rate	65.5% (76/116) of patients showed improvement
Han et al. (1986)	RCR	61 patients with diarrhea-predominant IBS	Three prescriptions including BGT	Mostly 0–3 weeks	Total effective rate	58% (15/26) of patients showed recovery and 31% (8/23) of patients showed improvement
Yoon (2001)	CR	One patient with collagenous colitis	Gami-BGT	3 months	Observed clinical symptom	• Frequency of defecation and stool condition returned to normal • Weight increased from 55 kg to 57.5 kg
Seo et al. (2004)	CR	One patient with Crohn’s disease	Gami-BGT	2 months (three times a day)	Observed clinical symptom	• Stool frequency and rectal bleeding normalized • Abdominal pain decreased • Anorexia improved until oral diet was possible

RCR, retrospective chart review; CR, case report; IBS, irritable bowel syndrome.

Chang et al. (1985) conducted a retrospective chart review of 116 patients with IBS treated at the Kyung Hee University Korean Medicine Hospital between 1984 and 1985. The primary complaints included lower abdominal discomfort (n = 62), insomnia (n = 55), and abdominal distension (n = 42). Among the eight different prescriptions dispensed, BGT was prescribed in 11.1% of the cases (18 patients). Seventy-six (65.5%) patients reported “improvement,” whereas 25 (21.6%) reported “no change.” Fifteen (12.9%) patients who did not revisit the clinic were labeled as “unknown.” Han et al. (1986) also conducted a chart review of 61 patients with diarrhea-predominant IBS treated at the same hospital between 1985 and 1986. The primary complaints included dyspepsia (n = 32) and abdominal pain (n = 29). Among the three prescriptions dispensed to the patients, BGT was prescribed in 26 cases. Fifteen of these patients “recovered,” whereas eight showed “improvement.”

Two case reports examined the effects of BGT on diarrhea. Yoon (Yoon, 2001) reported the case of a 52-year-old woman with collagenous colitis treated with gamma-BGT. Gami-BGT was administered for a period of 3 months for the management of chronic diarrhea that persisted for 15 years. The frequency of defecation and stool consistency returned to normal after treatment, with the weight of the patient increasing from 55 kg to 57.5 kg. Seo et al. (2004) reported the case of a 16-year-old boy diagnosed with Crohn’s who presented with persistent diarrhea, abdominal pain, rectal bleeding, and anorexia. Although surgery was recommended, the patient refused treatment and opted to continue medication. Gami-BGT was administered three times a day for a period of 2 months. The stool frequency and bloody stools normalized after treatment. Furthermore, the patient reported a decrease in abdominal pain and was able to resume an oral diet.

The findings of these studies indicate the clinical efficacy of BGT and its modified forms in the management of intestinal diseases, particularly diarrhea, including IBS and IBD.

### 3.3 Herbs

#### 3.3.1 Lonicera japonica Thunb

Ten *in vivo* studies, three *in vitro* studies, and one human RCT explored the effects of LJT. Three studies were conducted in both *in vivo* and *in vitro* settings (Table 3). Five studies (Lee et al., 2011; Park et al., 2013; Lv et al., 2021; Zhou et al., 2021; Chen et al., 2024) used a DSS-induced colitis model to investigate the effectiveness of LJT. Different concentrations of LJT extract were administered orally for a period of 7–21 d. Notably, symptom relief, alleviation of histological damage, and inhibition of inflammatory cytokines were observed in all five studies. Mechanisms underlying the effect of LJT on colitis were proposed in two of these studies. Park et al. (2013) proposed that LJT extract promotes recovery from colon damage through a cytokine response. LJT extract inhibit interferon (IFN)-γ and IL-17, which are representative T helper cell (Th)1 and Th17 cytokines, respectively. Furthermore, the extract also reduced the levels of Th1/Th17-related cytokines, such as IL-1β, 6, 12, TNF-α, in the colonic mucosa, but not those of IL-10, 23, or transforming growth factor (TGF)-β1. Thus, LJT attenuates colic damage by modulating the Th1/Th17 pathway rather than the regulatory T cell (T_reg_ cell) mechanism. Lv et al. (2021) reported that the LJT extract suppresses the protein expression of cleaved caspase-1, IL-1β, and IL-18 in the colonic macrophages by inhibiting the nucleotide-binding domain-like receptors family pyrin domain containing 3 (NLRP3) inflammasome. This effect was attributed to the enhancer of zeste homolog 2 (EZH2)/autophagy-related protein 5 (ATG5)-mediated autophagy regulation. The findings of these reports demonstrate that the LJT extract exerts anti-inflammatory effects on colitis through mechanisms at the cellular, protein, and gene levels.

**TABLE 3:** Studies of *Lonicera japonica Thunb.* on gastrointestinal function.

Study	Model	Species or cell	Inducer		Dose/Route/Regimen	Results
*in vivo*						
Lee et al. (2011)	Colitis	Male Balb/c mice	Oral administration of 4% DSS for 7 days		LJT water extract 1, 10, and 100 mg/kg p.o. for 12 days	LJT extract • Inhibited weight loss • Reduced the crypt injury and inflammation score • Decreased the serum amyloid A and MPO level
Park et al. (2013)	Colitis	Balb/c mice	Oral administration of 5% DSS for 7 days		LJT water extract 20, 100, and 500 mg/kg p.o. (two times every 7 days)	LJT extract • Inhibited weight loss and colon length shortening • Prevented histological damage • Downregulated IL-1β, TNF-α, IFN-γ, IL-6, IL-12, IL-17 • No significant changes were observed in IL-10, IL-23, TGF-β, and T_reg_ cell population LJT extract exhibited protective effects against DSS-induced colitis via the Th1/Th17 pathway, rather than T_reg_-related mechanisms.
Jung et al. (2014)	—	Male guinea pig (250–350 g)	—		LJT extract (GC 7101) 0.1, 0.5, 1, 5, and 10 mg/mL p.o. after testing	LJT extract • Increased the contraction amplitude of circular muscles in the antrum • Enhanced the migration length of charcoal in the small intestine • LJT extract enhanced gastrointestinal motility through cholinergic, antidopaminergic, and serotonergic mechanism.
	Delayed gastrointestinal motility		10^−6^ M Atropine p.o.			
			10^−8^ M Dopamine p.o.			
			10^−7^ M selective 5-HT4 receptor antagonist p.o.			
Nam et al. (2016)	—	Feline ESMC of Male cat (2.5–4 kg)	—		LJT extract (GC-7101) 0.1, 0.5, 1, 5, 10, and 20 μg/mL for 60 s before testing	LJT extract • Enhanced contractile responses of ESMCs • Restored tone and increased contractile responses in feline LES muscle strips • Accelerated gastric emptying and gastrointestinal transit The 5-HT3 and 5-HT4 receptor signaling pathways are involved in LJT-induced contraction.
	Decreased LES muscle tone	Feline LES muscle strip of Male cat (2.5–4 kg)	0.05 mM HCl for 60 s before challenge		LJT extract (GC-7101) 1 and 5 μg/mL	
	Tonic change of LES muscle		10^−7^ M cholinergic agonist (carbachol) for 15 min before challenge		LJT extract (GC-7101) 0.1, 0.5, 1, 5, and 10 μg/mL	
	Contractile response of LES muscle		Electric field stimulation (40 V, 1 ms, 4 Hz) for 10 s after challenge			
			10^−3^M NO inhibitor for 30 min and Electric field stimulation (40 V, 1 ms, 4 Hz) for 10 s			
	Delayed gastric emptying	Male SD rat (200–250 g)	—		LJT ethyl-alcohol extract (GC-7101) 1, 3, and 10 mg/kg	
			Loperamide (10 mg/kg) p.o.			
			Cisplatin (10 mg/kg) i.p.			
	Delayed gastrointestinal transit		—			
			Atropine (1 mg/kg) i.p.			
			Laparotomy of ileus			
Bang et al. (2019)	Gastritis	Male SD rat (180–200 g)	150 mM HCl in 60% ethanol p.o. 1 h before testing		LJT water extract (BST-104) 50, 100, and 200 mg/kg p.o. 1 h before challenge	LJT extract • Reduced gastric ulcer lesions • Increased gastric mucus contents (hexosamine, sialic acid, and PGE2) • Enhanced antioxidant activities (increased catalase, SOD, and GSH, but reduced MDA) • Suppressed TNF-α, IL-6, IL-1β, and NF-κB expression
	Peptic ulcer		50 l 30% acetic acid submucosal injection			
Minami and Makino (2020)	Digestive tract infection	Female C57BL/6 mice	Oral administration of 1× $10^{8}$ *Citrobacter rodentium* 1 day before testing		LJT water extract 1 and 2 g/kg/day p.o.	LJT extract • Increased survival rate • Decreased *Citrobacter rodentium* colonization • Upregulated TNF-α, IL-1β, and INF-γ
Lv et al. (2021)	Colitis	Female C57BL/6 mice (20–22 g)	Oral administration of 2.5% DSS for 7 days		LJT water extract 3, 10, and 30 mg/kg p.o. for 10 days	LJT extract • Reduced DAI scores (weight loss, diarrhea, bleeding) • Improved colon length shortening, splenomegaly, MPO activity • Alleviated mucosal damage, infiltration of inflammatory cells and loss of crypts • Inhibited cleaved-capase-1, IL-1β, and IL-18 expression in colonic macrophages
Zhou et al. (2021)	Colitis	Male Balb/c mice (18.9–21.9 g)	Oral administration of 5% DSS for 5 days		LJT polysaccharide 50, 100, and 150 mg/kg p.o. for 10 days	LJT polysaccharide • Increased spleen and thymus weight • Enhanced SIgA, serum concentrations of IL-2, TNF-α, and IFN-γ concentrations • Improved NK cell and CTL cytotoxicity • Enhanced intestinal probiotics and antagonized intestinal pathogenic bacteria • Inhibited spleen lymphocyte apoptosis
Sun et al. (2023)	Oxidative stress	Male ICR mice	Subcutaneous injection of D-galactose (200 mg/kg)		LJT polysaccharide 50 mg/kg and 100 mg/kg intragastrical injection for 8 weeks	LJT polysaccharide • Increased SOD, CAT, GSH-Px activity and Nrf2 expression • Decreased MDA levels • Restored gut microbiota by adjusting the *Firmicutes/Bacteroidetes* ratio and upregulating relative abundances of *Lactobabacillaceae* and *Bifidobacteriacesa* • LJT polysaccharide alleviated oxidative stress through the regulation of Nrf2 signaling.
Chen et al. (2024)	Colitis	Female C57BL/6 mice	Oral administration of 3.5% DSS for 7 days		LJT ethanol extract (200 mg/kg/day) for 21 days	LJT extract and its component chlorogenic acid • Decreased the DAI score, reduced colon mucosal injury, and inhibited colon length shortening • Reduced the serum IL-1β levels and increased colonic SOD, catalase activity, and serum GSH levels • Elevated the expression of Nrf2 and decreased TNF-α • Improved gut microbiota diversity and fecal SFCA production FMT of LJT-mediated gut microbiota alleviated disease symptoms of colitis.
*in vitro*						
Lee et al. (2011)	IL-6 synthesis	HT-29 human colon epithelial cell	Lipopolysaccharide		LJT water extract 5, 50, and 100 mcg/mL exposure for 72 h	LJT extract inhibited IL-6 synthesis
Lv et al. (2021)	Colitis	BMDMs from C57BL/6 mice			LJT water extract 3, 10, and 30 μmol/l	LJT extract • Inhibited protein expression of cleaved caspase-1 and IL-1β • Suppressed the secretion of IL-1β and IL-18 • Inhibited NLRP3 inflammasome assembly through lysosomal degradation of NLRP3 • Increased autophagosome production by upregulating ATG5 expression LJT extract alleviated colitis by inhibiting NLRP3 inflammasome, attributed to the regulation of EZH2/ATG5-mediated autophagy.
		Human monocytic THP-1 cell				
Chen et al. (2024)	—	Mouse leukemia virus-induced macrophage cell line RAW264.7	—		LJT ethanol extract 12.5, 25, 50, 100, and 200 mcg/mL for 2 h	LJT extract • Inhibited NO production • Increased serum catalase activity, serum GSH level, and colonic AOC • Improved gut microbial complexity and stability
Human						
Study	Study Type	Sample	Treatment	Duration of Administration	Evaluation	Result
Choi et al. (2020)	RCT	92 patients with FD	125 mg of LJT extract 300 mg or 300 mg placebo twice daily	8 weeks	GSRS NDI 8-OHdG (antioxidant) level Adverse effect	LJT extract • Improved the GSRS and NDI score • Reduced the 8-OHdG levels • No adverse events were reported

DSS, dextran sulfate sodium; LJT, *Lonicera japonica Thunb*.; p. o., per oral; i. p., intraperitoneal injection; MPO, myeloperoxidase assay; IL, interleukin; TNF, tumor necrosis factor; IFN, interferon; TGF, transforming growth factor; T_reg_, regulatory T cell; Th, Helper T cell; 5-HT, 5-hydroxytryptamine; ESMC, esophageal smooth muscle cell; LES, lower esophageal sphincter; HCl, Hydrogen chloride; NO, nitric oxide; sec, second; min, minute; h, hour; SD, sprague dawley; PGE2, Prostaglandin E2; SOD, superoxide dismutase; GSH, glutathione; MDA, malondialdehyde; NF-κB, Nuclear factor-κB; DAI, disease activity index; SIgA, Secretory immunoglobulin A; NK, cell, natural killer cell; CTL, cytotoxic lymphocyte; ICR, institute of cancer research; CAT, catalase; GSH-Px, Glutathione peroxidase; Nrf2, Nuclear factor erythroid 2-related factor 2; SCFAs, Short-chain fatty acids; FMT, fecal microbiota transplantation; BMDMs, Bone marrow-derived macrophages; NLRP3, nucleotide binding domain-like receptors family pyrin domain containing 3; ATG5, Autophagy-related protein 5; EZH2, Enhancer of zeste homolog 2; AOC, antioxidant capacity; RCT, randomized controlled trial; FD, functional dyspepsia; GSRS, gastrointestinal symptom rating scale; NDI, nepean dyspepsia index; 8-OHdG, 8-hydroxy-2′-deoxyguanosin.

The anti-inflammatory effects of LJT in gastritis and peptic ulcer models was demonstrated in the study by Bang et al. (2019). The administration of LJT extract alleviated gastric lesions, decreased the proinflammatory cytokine levels, decreased NF-κB expression, and increased gastric mucus production and antioxidant activity. Sun et al. (2023) reported that the extract exerts antioxidant effects by enhancing the activity of antioxidant enzymes such as superoxide dismutase (SOD), catalase (CAT), and glutathione peroxidase (GSH-Px), and by reducing the malondialdehyde (MDA) levels through the Nrf2 pathway.

The effects of LJT extract on *Citrobacter rodentium*-induced GI infection were investigated in the study conducted by Minami and Makino (Minami and Makino, 2020). The LJT extract increased the survival rates, enhanced macrophage phagocytic activity, increased the serum inflammatory cytokine levels, and suppressed bacterial colonization in mice. Thus, LJT may be a therapeutic agent for the management of infections of the human bacterial digestive tract.

Zhou et al. (2021) and Chen et al. (2024) evaluated the effect of LJT extract on the intestinal flora in DSS-induced colitis models. The administration of the LJT extract increased the levels of intestinal probiotics, such as *Bifidobacterium* and *Lactobacilli.* In contrast, it antagonized pathogenic bacteria, such as *Escherichia coli* and *Enterococcus* in a colitis model (Zhou et al., 2021). Similarly, Chen et al. (2024) reported an increase in the levels of *Lactobacillus* and *Romboutsia* following the administration of the LJT extract, along with a decrease in the levels of *Dubosiella*. The administration of the LJT extract also resulted in an increase in the production of total short-chain fatty acids (SCFAs) and gut microbiota-derived metabolites. Furthermore, the LJT extract exhibited significant correlations between specific bacteria and elevated fecal SCFA levels. Notably, fecal microbiota transplantation (FMT) from the LJT-treated group to the colitis group alleviated the symptoms and histological damage. Thus, FMT using LJT-mediated gut microbiota can improve colitis. Sun et al. (2023) also reported the regulatory effect of LJT on the intestinal microbiota in mice subjected to oxidative stress. Notably, LJT improved the microbial diversity, restored the *Firmicutes/Bacteroidetes* ratio, and increased the relative abundance of beneficial bacteria such as *Lactobacillaceae* and *Bifidobacteriaceae*, in addition to reversing the harmful changes induced by D-galactose. In other words, the LJT extract positively modulated the gut microbiota and promoted the production of metabolites, such as SCFAs.

The effect of LJT on GI motility was investigated in two studies (Jung et al., 2014; Nam et al., 2016). Jung et al. (2014) reported that the administration of the LJT extract resulted in a significant increase in the contraction amplitude of the circular muscle in the antrum and the migration percentage of charcoal along the small intestine. Atropine, dopamine, and a selective 5-HT4 receptor antagonist inhibited these effects, indicating that LJT exerts gastroprokinetic effects through cholinergic, dopaminergic, and serotonergic mechanisms. Nam et al. (2016), reported that the LJT extract enhanced the contractile responses of esophageal smooth muscle cells and lower esophageal sphincter (LES) muscle strips and accelerated the GI transit, thereby exerting a prokinetic effect on the GI tract. LJT also enhanced tonic responses in the LES muscle strips induced by carbachol, a cholinergic neurotransmitter that activates acetylcholine receptors. However, LJT-induced esophageal smooth muscle contraction was inhibited by pre-treatment with 5-HT3 and 5-HT4 receptor antagonists. This finding indicates that the cholinergic and serotonergic pathways are involved in LJT-induced contraction.

Only one clinical study was reviewed in the present study. This study by Choi et al. (2020) investigated the efficacy and safety of the administration of the LJT extract to patients with functional dyspepsia (FD). The study participants were divided into two groups: the LJT (46 patients) and placebo (46 patients) groups. A significant improvement in the symptoms and serum antioxidant levels was observed in the LJT group after 8 weeks of treatment. Notably, no treatment-related adverse events were reported.

In conclusion, the LJT extract exerted GI-protective effects in various GI dysfunction models, including IBD, gastritis, gastric ulcers, and GI infections, through its anti-inflammatory, antioxidant, and antibacterial properties. The gastroprokinetic effects exerted through the cholinergic and serotonergic pathways indicate its utility as a treatment for functional GI disorders such as FD.

#### 3.3.2 Atractylodes macrocephala Koidz

Twenty-four *in vivo* and 19 *in vitro* studies investigated the effects of AMK. Notably, six studies conducted both *in vivo* and *in vitro* experiments (Table 4). Eight *in vivo* studies (Feng et al., 2020; Li et al., 2021; Ren et al., 2021a; Kai et al., 2022; Yang et al., 2022; Cheng et al., 2023; Hu et al., 2023; Li et al., 2023) and two *in vitro* studies (Zong et al., 2021; Li et al., 2023) evaluated the effects of AMK on colitis. AMK inhibited weight loss, colon shortening, and overexpression of pro-inflammatory cytokines in all *in vivo* studies in addition to promoting the recovery of intestinal tissue damage, gut microbiota, and its metabolites. Yang et al. (2022) demonstrated the anti-inflammatory effects of AMK through the regulation of the Th17/T_reg_ cell balance in colitis, which may be mediated by inhibiting the IL-6/STAT3 signaling pathway, as indicated by the expression levels of inflammatory cytokines. Cheng et al. (2023) identified the mechanisms related to gut microbiota metabolism and discovered 56 AMK-related metabolites involved in maintaining intestinal homeostasis. Ascorbate and aldarate, arginine and proline, tryptophan, galactose, and amino sugar and nucleotide sugar metabolisms were the five most affected metabolic pathways. Hu et al. (2023) examined the effects of AMK on tryptophan metabolism and revealed that AMK treatment increased the levels of tryptophan metabolites, particularly those of aryl hydrocarbon receptor (AhR) ligands, in the feces and plasma. This finidng indicates that the beneficial effects of AMK on colitis are associated with the modulation of tryptophan metabolism. AMK polysaccharides decreased the apoptosis rate and increased the viability of intestinal epithelial cells in the *in vitro* study by Zong et al. (2021). This finding indicates that AMK enhanced the proliferation and survival of intestinal epithelial cells. AMK also upregulates the expression of tight junction proteins, thereby contributing to barrier formation. Microarray data indicates that the ITSN1-OT1 signaling pathway regulates the ability of AMK to restore intestinal barrier function by blocking the nuclear import of phosphorylated STAT2. Li et al. (2021) also reported that AMK enhanced the expression of tight junction proteins, such as ZO-1 and Occludin, indicating strengthened intestinal barrier integrity. These findings are comparable with those of the standard ulcerative colitis (UC) treatment with 5-Aminosalicylic acid. AMK alleviates colitis by strengthening barrier integrity through the enhanced expression of tight junction proteins. AMK and its polysaccharides demonstrated similar effects in TNBS-induced colitis, LPS-induced enteritis, and DSS-induced colitis models. Ren et al. (2021a) evaluated the therapeutic effects of Atractylenolide (ATL)-3, an AMK polysaccharide, on TNBS-induced colitis in mice. ATL-3 significantly alleviated colitis symptoms by reducing inflammation, lowering oxidative stress, and improving intestinal microbiota balance. Li et al. (2021) investigated the effects of AMK on LPS-induced enteritis and reported that AMK reduces intestinal inflammation by lowering the levels of inflammatory markers. Furthermore, it maintains the balance of the intestinal microbiota and improves the structural stability of the intestinal mucosa. The findings of these studies indicate that AMK exerts significant anti-inflammatory and protective effects against enteritis induced by various methods. Thus, AMK is a promising therapeutic agent for the treatment of IBD that supports overall gut health and stability.

**TABLE 4:** Studies of *Atractylodes macrocephala Koidz.* on gastrointestinal function.

Study	Model	Species or cell	Inducer	Dose/Route/Regimen	Results
*in vivo*					
Kim et al. (2005)	Allergic diarrhea	BALB/c mice	Intragastrical (50 mg) OVA administered for 3 weeks 1 week after subcutaneous immunization with 1mg OVA	AMK water extract (1 mg) p.o. for 3 weeks	AMK extract • Alleviated allergic diarrhea • Suppressed elevated total IgE and OVA-specific IgE levels • Increased the IFN-γ level
Xie et al. (2013)	—	Female ICR mice (18–22 g)	Subcutaneous injection of FMDV twice with 2-week intervals	AMK polysaccharide (0.05 g) for 4 days before challenge	AMK polysaccharide • Increased serum FMDV-specific IgG response, total SIgA concentration, area of IgA^+^ cells, and number of IELs in the duodenum • Elevated the mRNA expression of TGF-β, IL-6, TNF-α in the duodenum
Wang et al. (2014)	Disordered intestinal flora	Male SD rat (190–200 g)	Oral administration of CAE 10 g/kg (2 times every 10 days)	AMK polysaccharide solution 0.035 and 0.105 g/kg for 10 days	AMK polysaccharide • Activated growth and improved the structure of intestinal flora • Alleviated watery stool • Increased body weight and vigor
Feng et al. (2019)	Colorectal cancer	TLR4 KO C57BL/6 or WT mice	Injection of MC38 colorectal cancer cell in flank	Intraperitoneal injection of AMK polysaccharide 500 mg/kg three times a week for 2 weeks	AMK polysaccharide • Suppressed tumor volume in WT mice • Extended survival time in WT mice • Reduced the TNF-α, IFN-λ, and IL-6 levels in WT mice AMK polysaccharide induced anti-tumor immunity by triggering TLR4-dependenet macrophage activation
Feng et al. (2020)	Colitis	SPF-free-grade male C57BL/6J mice (18–22 g)	Oral administration of 2.5% DSS for 7 days	Intragastrical AMK polysaccharide 10, 20, and 40 mg/kg for 3 days	AMK polysaccharide • Inhibited body weight loss and colon length shortening • Alleviated rectal bleeding and diarrhea • Recovered histological damage of the intestine • Reduced TNF-α, IL-18, and IL-1β levels in the colon • Recovered gut microbiota diversity and metabolites • Modulated SCFA production
Li et al. (2021)	Enteritis	Goslings (*Anser cygnoides*)	LPS (2 mg/kg)	AMK polysaccharide 400 mg/kg p.o. for 3 days	AMK polysaccharide • Protected intestinal morphology and maintained villi structure • Reduced the CRP, IL-1β, IL-6, and TNF-α levels • Maintained intestinal flora stability by regulating the abundance of *Romboutsia* • Increased the Occludin and ZO-1 level
Ren et al. (2021a)	Colitis	Male C57BL/6 mice (22–25 g)	TNBS (150 mg/kg) administered rectally for 7 days	Atractylenolide Ⅲ 5, 10, 20 mg/kg p.o. for 7 days	• Atractylenolide Ⅲ • Decreased body weight loss and the DAI score • Reduced the MPO, IL-1β, and TNF-α levels • Lowered oxidative stress markers (MDA and ROS) and enhanced antioxidants (CAT, SOD, and GSH-Px) • Improved intestinal flora balance, increasing the *Lactobacillus* count. • Reduced FPR1 and Nrf2 protein expression levels
Amin et al. (2022)	Gastritis	Male SD rat (200 g)	HCl Ethanol 1 mL/200 g	Intragastrical AMK ethanol extract 35 mg/kg (two times every 3 days)	AMK extract • Decreased gastric tissue damage • Prevented immune cell recruitment
Jia et al. (2022)	Constipation	Male SD rat	Loperamide 3 mg/kg	Intragastrical administration of AMK water extract, ethanol extract, polysaccharide (8.64 g/kg) for 14 days	AMK water extract • Increased fecal water content and fecal output • Increased intestinal motility • AMK polysaccharide • Elevated the MTL levels and decreased the VIP levels in plasma • Enhanced mucosal barrier function by increasing MUC2 and ZO-1 protein expression in colonic tissue
Kai et al. (2022)	Colitis	C57BL/6 mice (17.24–19.14 g)	Oral administration of 3% DSS for 7 days	Intragastrical AMK polysaccharide (100 mg/kg) for 2 weeks before challenge	AMK polysaccharide • Inhibited body weight loss, colon length shortening, and colonic damage • Increased Mucin-2 and Claudin-1 expression • Decreased colonic neutrophil infiltration • Reduced the TNF- α, IL-6, and IL-1β levels • Modulated overall intestinal microbiota richness and diversity
Yang et al. (2022)	Colitis	SPF-grade male C57BL/6J mice (18–22 g)	Oral administration of 3% DSS for 7 days	AMK polysaccharide 100, 200, and 400 mg/kg for 7 days	AMK polysaccharide • Inhibited body weight loss and colon length shortening • Reduced the DAI score, histopathological score, and MPO activity • Decreased the TNF-α, IL-1β, IL-18, and IL-23 levels • Increased tight junction protein expression • Maintained Th17/T_reg_ cell homeostasis
Wang et al. (2022)	Diarrhea	Kunming strain mice (18–22 g)	*E. coli* (0.5 mL/time/day) i.p. injection for 7 days	Oral administration of AMK polysaccharide 1.2, 3.6, and 6 mg/mL 24 h before challenge	AMK polysaccharide • Improved mental state and appetite • Alleviated duodenal mucosa histology • Reduced iIELs in the duodenum and ileum • Increased iIELs in the jejunum • Decreased the IL-6 and TNF-α levels in the duodenum
Zhang et al. (2022)	Colorectal cancer	SPF-male BALB/c-nu mice (18–20 g)	Subcutaneously inoculated HCT-116 tumor xenografts	Intragastrical ATL-Ⅲ 50, 100, and 200 mg/kg for 4 weeks	ATL-Ⅲ from AMK • Inhibited tumor growth • Reduced tumor volume and weight • Enhanced the expression of p53 and apoptotic markers
Cheng et al. (2023)	Colitis	Male C57BL/6 mice (18–22 g)	Oral administration of 2.5% DSS for 8 days	AMK volatile oil (0.8 μl/10 g) BW for 11 days	AMK volatile oil • Alleviated bloody diarrhea, colon damage, and inflammation • Decreased harmful bacteria and enriched beneficial bacteria of the gut AMK volatile oil altered the gut microbiota metabolism by regulating 56 gut microbiota metabolites involved in 102 KEGG pathways, including tryptophan metabolism, taurine and hypotaurine metabolism, sphingolipid signaling pathway, pyrimidine metabolism, and purine metabolism.
Hu et al. (2023)	Colitis	Male C57BL/6J mice (18–22 g)	Oral administration of 2.5% DSS for 7 days	Intragastrical AMK polysaccharides (20 mg/kg BW) for 3 days before challenge	AMK polysaccharide • Inhibited body weight loss and colon length shortening • Restored colon tissue architecture • Normalized goblet cells • Increased fecal and plasma AhR ligands (Iald, ILA, PA, and Trp) and plasma Ser and 5-HTAA Mechanism of AMK polysaccharide in treating ulcerative colitis is associated with increasing AhR ligands in the feces and plasma.
Li et al. (2023)	Colitis	Female Balb/c mice (18–20 g)	Oral administration of 3% DSS for 7 days	AMK essential oil 50 mg/kg p.o. for 7 days	AMK essential oil • Reduced inflammation and oxidative stress • Lowered the TNF-α and IL-6 levels • Improved UC symptoms, thereby reducing weight loss and colon shortening • Enhanced tight junction proteins (ZO-1 and Occludin), aiding intestinal barrier integrity
Yang et al. (2023)	Constipation	Male SPF-grade ICR mice (16–20 g)	Intragastrical Senna leaf decoction for 7 days and 5–10g of raw rice for 8 days	AMK polysaccharide 0.5mL (0.021g/mL) for 7 days	AMK polysaccharide • Increased the fecal water content and fecal pallets • Normalized gastrointestinal transit rate • Altered 5-HT and CgA expression • Increased fecal SCFAs content • Alleviated dysbiosis of the gut microbiota AMK polysaccharide modulated the intestinal flora by targeting the abundance of key strains and metabolic pathways, mainly tryptophan metabolism, unsaturated fatty acid biosynthesis, primary bile acid metabolism.
Zeng et al. (2023)	Gastric ulcer	SPF-grade male SD rat (180–200 g)	Intragastrical 95% ethanol 1 mL/100 g for 14 days	Intragastrical 2.16 g/kg raw AMK; 2.16 g/kg bran-fried AMK; 1.08, 2.16, and 4.32 g/kg honey-bran-fried AMK for 15 days	AMK, especially honey-bran-fried AMK • Decreased the ulcer index and ulcer area • Increased levels of SOD and GSH-Px • Reduced the MDA, IL-1β, IL-6, TNF-α, IL-1β, MMP-9, TIMP-1, NF-κB-protein levels • Regulated extracellular matrix degradation • Normalized intestinal flora Anti-ulcer and anti-inflammatory effect of AMK is related to NF-κB-MMP-9/TIMP-1 signaling pathway.
Zhai et al. (2023)	Gastric ulcer	SPF-grade male SD rat (190–210 g)	Intragastrical 95% ethanol 1 mL/100 g	Carbon dots of Charred AMK 2.24, 6.72, and 26.88 mg/kg	Carbon dots of Charred AMK • Alleviated gastric ulcer • Improved bleeding and inflammatory cell infiltration of the stomach • Decreased the IL-6, IL-1β, TNF-α, and MDA levels • Increased the IL-10, PGE2, and MUC5AG levels • Inhibited H^+^-K^+^-ATPase and pepsin activity • Improved microbial diversity Carbon dots of Charred AMK ameliorate gastric ulcer through the inhibition of the NF-κB/NLRP3 axis.
Chenxing et al. (2024)	Diarrhea	Pregnant SPF-grade ICR mice (20–27 g)	Intragastrical 0.4 mL, 1 g/mL Senna Folium for 15 weeks	AMK water extract 0.25, 0.50, and 1.00 g/kg	AMK water extract • Alleviated diarrhea • Increased body weight • Increased gastric emptying rate, small intestinal propulsion rate, and gastrointestinal hormone levels (serum level of motilin, ghrelin, growth hormone, α-amylase) • Improved inflammatory infiltration, intestinal glands, mucin content, but decreased goblet cell destruction • Reduced protein and mRNA level of claudin-2 • Increased expression of AQP3, AQP4 and AQP8 in colonic tissue
Choi et al. (2024b)	Gastric adenocarcinoma	Female NSG mice	Subcutaneously inoculated AGS-iRFP cells in flank	AMK water extract 10 and 50 mg/kg	AMK extract inhibited tumor growth and iRFP signal
Qin et al. (2024)	Constipation	Male SD rat (160–200 g)	Intragastrical (3 mg/kg/day) loperamide hydrochloride twice per day for 14 days	AMK water extract 2.16, 4.32, and 8.64 g/kg	AMK extract • Increased fecal water content, Bristol score, and gastrointestinal transit rate • Recovery of the damaged intestinal tissue • Regulated gut neurotransmitters in the serum (vasoactive intestinal peptide, somatostatin, dopamine, motilin, gastrin, 5-HT) • Normalized abnormal levels of urine tryptophan metabolites (4,6-dihydroxyquinoline, indole, 4,8-dihydroxyquinoline, 5-HT, and kynurenic acid) • Normalized abnormal expression of rate-limiting enzyme involving in tryptophan metabolism (TPH, MAO, and IDO) Laxative effect of AMK regulates the disturbance of tryptophan metabolism.
Choi et al. (2024a)	IBS	Male C57/BL6 mice	Zymosan (30 mg/mL) 0.1mL administration through colon for 3 days	AMK extract 250 and 500 mg/kg p.o. for 12 days	AMK extract • Reduced inflammation in colonic tissues • Normalized colon length and body weight • Improved stool consistency • Modulated ion channels (TRPV1, NaV1.5, NaV1.7), reducing visceral hypersensitivity
*in vitro*					
Kim et al. (2005)	—	Splenocyte	—	10 μg/mL AMK protein sample for 48 h	AMK protein sample • Increased the levels of total IgG, IFN-γ, IL-2 • Stimulated splenocyte proliferation
Ma et al. (2014)	Gastric cancer	Human gastric cell lines (MGC-803, HGC-27, and MKN-45)	—	ATL-1 0–100 μM for 24–72 h	ATL-1 from AMK • Inhibited cell viability and induced apoptosis • Inactivated Notch signaling (Notch1, Jagged1, Hes1, and Hey1) • Reduced the self-renewal and colony formation abilities of GCSLCs • Decreased the expression of CD44 • ATL-1 can potentially inhibit cancer cell proliferation and induce apoptosis through inactivating Notch pathway.
Song et al. (2014)	Cell migration	IEC-6 cell	Wound induced by a single-edged razor blade	AMK methanol extract 50, 100, and 200 μg/mL	AMK methanol extract • Increased the cellular polyamine content, membrane hyperpolarization, [Ca^2+^] cyt, and cell migration • Reversed the inhibitory effects of DFMO on polyamines, membrane potential, and [Ca^2+^] cyt • Upregulated Kv1.1 mRNA and protein expression • AMK extract stimulated migration of IEC-6 cells through polyamine-Kv1.1 channel signaling.
Shim et al. (2015)	Colon cancer	SW-480	—	Atractylochromene (20 μg/mL) for 6–72 h	Atractylochromene from AMK • Inhibited β-catenin nuclear translocation • Decreased levels of cyclin D1, target gene of β-catenin, and galectin-3, and β-catenin nuclear translocation modulator • Suppressed cell viability • Atractylochromene inhibits the Wnt/β-catenin signaling pathway through modulation of the nuclear translocation of β-catenin and galectin-3 in colon cancer cells.
Song et al. (2015)	Cell migration	IEC-6 cell	Wound induced by a single-edged razor blade	AMK methanol extract 50, 100, and 200 g/l	AMK methanol extract • Increased cellular polyamine levels, Rho mRNA, and proteins expression • Enhanced cell migration via increased non-muscle myosin 2 protein and stress fiber formation AMK promoted the migration of IEC-6 cells through a polyamine-dependent pathway
Song et al. (2017)	IEC injury	IEC-6 cell	Wound-induced by a scratch with a gel-loading microtip	ATL-1 5 and 10 µM for 24 h	ATL-1 from AMK • Promoted cell migration and proliferation • Increased polyamine content • Elevated the cytosolic free Ca^2+^ concentration • Enhanced mRNA/protein expression of TRPC1 and PLC-γ1 • ATL-1 stimulates intestinal epithelial cell migration and proliferation via the polyamine-mediated Ca^2+^ signaling pathway
Tian and Yu (2017)	Gastric carcinoma	HGC-27, AGS	—	ATL-2 50, 100, 200, 400, and 10 µM for 24, 48, and 72 h	ATL-2 from AMK • Inhibited proliferation and induced apoptosis • Reduced cell motility • Downregulated p-Akt and p-ERK • Increased Bax/Bcl-2 ratio • ATL-2 exerted significant anti-tumor effects on the gastric carcinoma cells by modulating Akt/ERK signaling pathway.
Feng et al. (2019)	Colorectal cancer	MC38 cell derived from C57BL/6 murine colon adenocarcinoma	—	AMK polysaccharide	AMK polysaccharide • Enhanced phagocytosis by BMDMs • Activated expression and secretion of immunomodulatory factors (IL-6, IFN-λ, TNF-α, and NO) in BMDMs through TLR4 and MyD88 The TLR4/MyD88 pathway plays a vital role in anti-cancer effects of AMK.
		CT26 cell derived from BALB/c murine colon carcinoma			
		RAW264.7 cell			
Chan et al. (2020)	Colon adenocarcinoma	HT-29	—	ATL-1 10, 20, 40, 80, 100, and 200 μM for 24, 48, and 72 h	ATL-1 from AMK • Decreased cell viability • Promoted DNA fragmentation without necrotic effects. • Increased expression levels of cleaved caspase-3, -7, -8, -9, and cleaved PARP • Increased protein expression levels of anti-survival Bcl-2 family proteins and decreased Bcl-2 • ATL-1 showed anti-cancer effect through the activation of caspases and pro-apoptotic Bcl-2 family proteins, and the mitochondrial-dependent pathway is involved.
Yang et al. (2020)	Chronic gastritis	RAW264.7 cell	LPS 1 μg/mL for 24 h	ATL-1 20, 40, and 60 μM/L for 24 h after challenge	ATL-1 from AMK • Decreased IL-6 production • Decreased IL-6 and IL-1β mRNA expression
Ren et al. (2021b)	IEC injury	IEC-6	—	ATL-3 10, 20, and 40 μM for 24 and 48 h	ATL-3 from AMK • Promoted cell proliferation and migration • Increased the intracellular Ca^2+^ levels through the Ca^2+^ signaling pathway • Enhanced the expression of STIM1, TRPC1, PLC-γ1, and RhoA proteins • Upregulated anti-inflammatory cytokines IL-2, IL-10, and ODC • ATL-3 promoted intestinal epithelial repair through activating the Ca^2+^ pathway
Zong et al. (2021)	IECs injury	IPEC-J2	3% DSS	AMK polysaccharide RAMPtp 2–200 μg/mL	AMK polysaccharide RAMPtp • Reduced cell viability and apoptosis • Increased the expression of intestinal barrier proteins (Occludin, claudin-1, ZO-1) • Decreased the IL-6, TNF-α, and IL-1β levels • RAMPtp restored intestinal barrier dysfunction via IncRNA ITSN1-OT1 signaling pathway, blocking the nuclear import of phosphorylated STAT2
Amin et al. (2022)	Inflammation	RAW264.7 cell	LPS 1 μg/mL 5, 15, 30, 60 m, 22 h	AMK ethanol extract (50 μg/mL) for 1 h after challenge	AMK extract • Suppressed NO and PGE2 production • Reduced iNOS and COX-2 mRNA expression • Inhibited NF-κB p50 subunit nuclear translocation and NF-κB-stimulated LRA activity • Decreased phosphorylated IκBα and AKT protein levels AMK extract exhibited anti-inflammatory effects through the AKT/IκBα/NF-κB signaling pathway.
Sun et al. (2022)	Colorectal cancer	HCT-116 cell	—	ATL-1 25, 50, 100, and 200µM for 72 h	ATL-1 from AMK • Inhibited cell proliferation, migration, and invasion • Downregulated PDK1 and inhibited FoxO1 phosphorylation • Decreased Bcl-2 and increased Bax expression • Decreased MMP2 and vimentin and increased E-cadherin expression • ATL-1 inhibited the malignant development of cancer cells and increased oxaliplatin sensitivity by decreasing PDK1 and inhibiting FoxO1 phosphorylation.
Zhang et al. (2022)	Colorectal cancer	HCT-116 cell	—	ATL-3 25, 50, 100, and 200 μM for 24 h	ATL-3 from AMK • Induced apoptosis • Increased Bax, caspase-9, and caspase-3 activation • Inhibited Bcl-2 expression • ATL-3 induced apoptosis via Bax/Bcl-2 pathway
Chen et al. (2023)	Colon cancer	CT-26 cell	—	AMK polysaccharide 100 μM	AMK polysaccharide • Exhibited cytotoxicity against colon cancer cell • Promoted apoptosis of colon cancer cell
Xu et al. (2023)	Oxidative stress	NCM460	H_2_O_2_ 1000, 2000, and 4000 μM	ATL-1 5 μg/mL	ATL-1 from AMK • Increased cell viability and reduced apoptosis • Inhibited up-regulated miR-34a-5p expression • Promoted basal glucose metabolism • ATL-I treatment reversed miR-34a-5p-inhibited glucose metabolism and exacerbated colonic mucosal epithelial cell dysfunction under oxidative stress by modulating the miR-34a-5p-LDHA pathway.
Lemons et al. (2023)	—	Human adult gut microbiome	—	3 g/L AMK extract for 48 h	AMK extract • Decreased Bacteroidetes phylum • Increased beneficial *Bifidobacterium spp.* and SCFAs
Li et al. (2023)	inflammation	RAW264.7 cell	LPS 10 ng/mL for 12 h	AMK essential oil 1, 5, 25, and 125 μg/mL for 8 h	AMK essential oil • Decreased TNF-α and IL-6 levels • Lowered ROS and MDA, increased SOD and GSH
Choi et al. (2024b)	Gastric cancer	AGS cell	—	AMK extract 50, 100, 150, and 200 μg/mL	AMK extract • Reduced AGS cell growth • Increased cell cycle sub-G1 phase • Induced apoptosis via ROS generation • Upregulated mitochondrial depolarization and reduced TMRM-positive fluorescence • Increased cleavage of pro-Caspase-9, PARP, and caspase-3 • Altered Bax/Bcl-2 ratio and reduced the level of Bcl-2 and Bcl-xL levels • Increased p38 and JNK phosphorylation and reduced the phosphorylation levels of ERK and AKT • Inhibited cell migration AMK extract induced apoptosis in gastric cancer cell through intrinsic mitochondrial pathway.

OVA, ovalbumin; AMK, Atractylodes macrocephala Koidz.; p. o., per oral; Ig, Immunoglobulin; INF, interferon; ICR, institute of cancer research; FMDV, Foot-and-mouth disease vaccine; SIgA, Secretory immunoglobulin A; IELs, Intestinal intraepithelial lymphocytes; mRNA, messenger RNA; TGF-β, Transforming growth factor β; IL, interleukin, TNF-α: Tumor necrosis factor α, SD: sprague dawely, CAE: Cassia angustifolia Vahl (Senna) extract, TLR4: Toll-like receptor 4, KO: knock out, WT: wild type, SPF: Specific-pathogen-free, DSS: dextran sulfate sodium, SCFAs: Short-chain fatty acids, LPS: lipopolysaccharide, CRP: C-reactive protein, ZO-1: zonula occludens-1, TNBS: 2,4,6-trinitrobenzenesulfonic acid, DAI: disease activity index, MPO: myeloperoxidase, MDA: malondialdehyde, ROS: reactive oxygen species, CAT: catalase, SOD: superoxide dismutase, GSH-Px: Glutathione peroxidase, FPR1: Formyl peptide receptor 1, Nrf2: Nuclear respiratory factor 2, HCl: Hydrogen chloride, MTL: motilin, VIP: vasoactive intestinal peptide, MUC2: Mucoprotein 2, Th: Helper T cell, Treg: regulatory T cell, ATL: atractylenolide, i. p.: intraperitoneal, h: hour, iIELs: intestinal intraepithelial lymphocytes, BW: body weight, KEGG: kyoto encyclopedia of genes and genomes, IAld: Indole-3-aldehyde, ILA: Indole-3-lactic acid, AhR: aryl hydrocarbon receptor, PA: picolinic acid, Trp: Tryptophan, Ser: Serotonin, 5-HTAA: 5-hydroxy-tryptophan, 5-HT: 5-hydroxytryptamine, CgA: Chromogranin A, MMP-9: Matrix metalloproteinase-9, TIMP-1: Tissue inhibitor of meralloproteinase-1, NF-κB: Nuclear transcription factor-κB, PGE2: Prostaglandin E2, AQP: aquaporin, NSG: NOD SCID, gamma immunodeficient, TPH: tryptophan hydroxylase, MAO: monoamine oxidase, IDO: Indoleamine-2, 3-dioxygenase, IBS: irritable bowel disease, GCSLCs: gastric cancer stem-like cells, IEC: intestinal epithelial cell, [Ca2+]cyt: cytosolic free [Ca2+] concentration, DFMO: difluoromethylornithine, Kv 1.1: polyamine-voltage-gated K+ channel a-subunit 1.1, TRPC1: canonical transient receptor potential-1, PLC: Phospholipase C, p-Akt: phosphorylated-protein kinase B, p-ERK: phosphorylated-ERK, Bax: Bcl-2-associated X protein, Bcl-2: B-cell lymphoma-2, AKT: Protein kinase B, ERK: extracellular signal-regulated kinase, BMDMs: Bone marrow-derived macrophages, NO: nitric oxide, MyD88: Myeloid differentiation primary response gene 88, PARP: Poly ADP, ribose polymerase, STIM1: Stromal interaction molecule 1, TRPC1: Transient receptor potential 1, PLC-γ1: Phospholipase C-γ1, ODC: ornithine decarboxylase, IPEC-J2: porcine intestinal epithelial cell, lncRNA: Long non-coding RNA, STAT2: Signal transducer and activator of transcription 2, iNOS: inducible NO, synthase, COX-2: Cyclolxygenase-2, LRA: luciferase reporter gene assays, IκB: Inhibitor of NF-κB, PDK1: Pyruvate dehydrogenase kinase 1, FoxO1: Forkhead box protein O1, H_2_O_2_: hydrogen peroxide, TMRM: tetramethylrhodamine methyl ester, Bcl-xL: B-cell lymphoma-extra-large, p38: p38 mitogen-activated protein kinases, JNK: c-Jun N-terminal kinase.

Twenty studies examined the effect of AMK in digestive tract disease models, including diarrhea, constipation, IBS, gastritis, gastric ulcer, and GI cancer. The effects of AMK extract on diarrhea were evaluated in three studies (Kim et al., 2005; Wang et al., 2022; Chenxing et al., 2024). AMK extract alleviated symptoms and reduced the levels of inflammatory cytokines in all three studies. Wang et al. (2022) demonstrated the effects of AMK polysaccharides on improving watery stools and the restoration of the intestinal flora in a senna-induced diarrhea model. These findings suggest that AMK polysaccharides has a positive effect on gut microbiota balance, which is essential for GI health. Chenxing et al. (2024) reported that the administration of AMK extract significantly improved mucosal integrity, increased GI hormone levels, and enhanced the intestinal propulsion rate. Thus, the AMK extract may strengthen the structural and functional health of the gastrointestinal system, thereby contributing to diarrhea relief. Furthermore, AMK extract stimulated the expression of aquaporins, epithelial tight junction proteins, and mRNA, which play crucial roles in water reabsorption from the colon and reinforcement of intestinal epithelial integrity. These effects underscore the role of AMK in promoting water balance and supporting the epithelial barrier in the intestine, contributing to its anti-diarrheal properties.

Three studies (Jia et al., 2022; Yang et al., 2023; Qin et al., 2024) investigated the effects of AMK extract in a constipation model, with a focus on symptom relief and the underlying biochemical pathways. AMK extract alleviated constipation by increasing fecal water content and enhancing the GI transit rate in all three studies. Analysis of the effect of AMK on tryptophan metabolism revealed its role in regulating GI hormone and gut neurotransmitter levels, including 5-HT. Yang et al. (2023) reported that AMK-treated mice exhibited an increase in the count of beneficial gut flora and SCFAs. Furthermore, they reported significant changes in 30 metabolites and 15 metabolic pathways, including tryptophan metabolism, unsaturated fatty acid biosynthesis, and primary bile acid biosynthesis. These metabolites produced by the intestinal microorganisms stimulate tryptophan metabolism, leading to the synthesis of 5-HT, which is associated with improved gut motility. Qin et al. (2024) identified 14 altered metabolites and four metabolic pathways (i.e., tryptophan metabolism, taurine and hypotaurine metabolism, tyrosine metabolism, and primary bile acid biosynthesis) regulated by AMK treatment that were correlated with constipation indicators. This study also highlighted the ability of AMK to modulate key enzymes in the tryptophan metabolic pathway, such as tryptophan hydroxylase, monoamine oxidase, and indoleamine-2,3-dioxygenase. These findings indicate that tryptophan metabolism plays a crucial role in relieving constipation through AMK. Collectively, the findings of these studies provide evidence that AMK extract may alleviate constipation by modulating the gut microbiota, metabolic pathways, and neurotransmitter levels, especially through the regulation of the metabolism of tryptophan, thereby contributing to increased 5-HT synthesis and enhanced GI function.

Choi et al. (2024a) used a mouse model of zymosan-induced IBS to explore the effects of AMK on IBS and revealed that AMK effectively reduced colonic inflammation, normalized colon length, and improved stool consistency. Thus, AMK yielded results comparable with those of established IBS treatments such as amitriptyline and sulfasalazine. The findings of this study suggest the potential of AMK as a therapeutic agent for IBS, primarily through the modulation of ion channels, particularly TRPV1, which is involved in reducing the visceral hypersensitivity associated with IBS.

Amin et al. (2022) and Yang et al. (2020) investigated the effects of AMK in a gastritis model, with a focus on its anti-inflammatory and protective mechanisms. Yang et al. (2020) examined ATL, a polysaccharide component of AMK, and reported its ability to reduce the expression of pro-inflammatory cytokines IL-6 and IL-1β, suggesting a targeted anti-inflammatory action. Amin et al. (2022) conducted *in vivo* and *in vitro* studies. The *in vivo* study revealed that the administration of the AMK extract decreased gastric tissue damage and immune cell infiltration, highlighting its protective role against gastric injury. The *in vitro* study revealed that the AMK extract inhibited the upregulation of inflammatory mediators, including nitric oxide (NO) and prostaglandin E2 (PGE2), and downregulated the expression of related inflammatory genes and proteins. Furthermore, AMK extract also suppressed nuclear translocation of the NF-κB subunit p50 and increased the phosphorylation of IκBα and Akt. These findings indicate that its anti-inflammatory and antioxidant effects are mediated through the modulation of the Akt/IκBα/NF-κB signaling pathway.

Two studies (Zeng et al., 2023; Zhai et al., 2023) investigated the effects of AMK in an ethanol-induced gastric ulcer model and demonstrated the ability of AMK to alleviate gastric ulcers, reduce inflammatory markers, and improve the balance of intestinal flora. Zeng et al. (2023) measured the levels of oxidative stress markers and reported that AMK reduced the MDA levels, a marker of oxidative stress, and increased the levels of antioxidant enzymes such as SOD and GSH-Px. Furthermore, AMK normalized the expression of matrix metalloproteinase-9 (MMP-9), tissue inhibitor of metalloproteinase-1 (TIMP-1), and NF-κB proteins. These findings indicate that AMK alleviates ulcers by restoring balance in the NF-κB-MMP-9/TIMP-1 pathway. Zhai et al. (2023) explored the mechanism of the anti-ulcer effects of AMK and revealed that it inhibited the activation of the NLRP3 inflammasome, a key trigger of NF-κB activation that leads to inflammatory damage. The inhibition of the NF-κB/NLRP3 axis was central to the protective effects of AMK against ethanol-induced gastric ulcers. Pepsin activity, H^+^-K^+^-ATPase enzymatic activity (an indicator of gastric acid secretion), and the levels of malondialdehyde (MDA) and the mucosal protective factors PGE2 and MUC5AC were also quantified. AMK protected the gastric mucosa by reducing inflammation and oxidative stress, inhibiting H^+^-K^+^-ATPase and pepsin activities, and enhancing the levels of PGE2 and mucin. Collectively, these findings suggest that the AMK extract can potentially treat gastritis and gastric ulcers by modulating oxidative stress, inflammatory pathways, and gastric mucosa protective mechanisms, particularly through the NF-κB-related pathways.

The anti-cancer effects of AMK were examined by nine studies (Ma et al., 2014; Shim et al., 2015; Tian and Yu, 2017; Feng et al., 2019; Chan et al., 2020; Sun et al., 2022; Zhang et al., 2022; Chen et al., 2023; Choi et al., 2024b) using colon, colorectal, and gastric cancer models. Chen et al. (2023) explored the cytotoxic and apoptotic effects of AMK polysaccharides on colon cancer cells. Shim et al. (2015) explored the anti-cancer properties of Atractylochromene, a compound present in AMK, with a specific target on colon cancer through the inhibition of the Wnt/β-catenin signaling pathway. Atractylochromene suppressed the nuclear translocation of β-catenin and galectin-3 in SW-480 colon cancer cells, thereby reducing the expression of cyclin D1, a gene involved in cancer cell proliferation, ultimately resulting in decreased growth of the cancer cells. Chan et al. (2020) investigated the anti-tumor potential of ATL-1 in the human colon adenocarcinoma cell line HT-29 and revealed that ATL-1 induced apoptosis through a mitochondria-dependent pathway by modulating pro-apoptotic (Bax/Bak) and anti-apoptotic (Bcl-2) proteins. This resulted in the activation of a caspase cascade essential for apoptosis without inducing necrosis. The findings of these studies underscore the potential of AMK in colon cancer treatment through the inhibition of cancer cell proliferation and the promotion of mitochondria-mediated apoptosis. Feng et al. (2019) investigated the role of AMK polysaccharides in colorectal cancer and reported that AMK enhances the phagocytosis of cancer cells by promoting the migration of bone marrow-derived macrophages (BMDMs) and increased mRNA expression of inflammatory cytokines and NO. Furthermore, AMK specifically activates toll-like receptor 4 (TLR4), which plays a pivotal role in the activation of macrophages. Notably, TLR4 and MyD88 inhibitors suppressed the anti-tumor effect of AMK, indicating that AMK promotes anti-cancer immunity via the TLR4/MyD88 pathway. This finding is further supported by the *in vivo* results in TLR4-deficient mice. Sun et al. (2022) reported that the application of ATL-1 to HCT-116 colorectal cancer cells reduced cell viability and motility, induced apoptosis, and enhanced oxaliplatin chemotherapy efficacy through the downregulation of the PDK1/FoxO1 pathway. This led to increased apoptosis and decreased tumor-promoting behaviors. *In vitro* and *in vivo* models were used by Zhang et al. (2022) to evaluate the anticancer effects of ATL-3 on colorectal cancer. ATL-3 significantly inhibited tumor growth and upregulated the expression of apoptotic markers, such as p53, in the *in vivo* model. ATL-3 induced apoptosis in cancer cells through the Bax/Bcl-2 signaling pathway and by activating caspases in the *in vitro* model. Thus, AMK and its components, ATL-1 and ATL-3, exhibit promising anti-tumor effects in colorectal cancer by leveraging immune activation and apoptotic modulation, thereby potentially enhancing chemotherapy outcomes. Choi et al. (2024b) observed that the AMK extract induced apoptosis by reducing cell growth and increasing ROS generation, revealing mitochondrial pathway involvement through altered membrane potential, caspase activity, and B-cell lymphoma protein levels. Ma et al. (2014) investigated the effect of ATL-1 on gastric cancer cells, with a particular focus on cancer stem cell characteristics. ATL-1 inhibited gastric cancer cell proliferation and induced apoptosis by targeting the Notch signaling pathway, a critical regulator of cancer progression. Notably, ATL-1 decreased the expression of Notch1 and associated proteins such as Jagged1, Hes1, and Hey1, thereby reducing cancer cell growth. ATL-1 also specifically targeted gastric cancer stem-like cells (GCSLCs) by impairing their colony formation and self-renewal capabilities, suggesting its potential to serve as a therapeutic agent by targeting pathways essential for tumor growth and maintenance. Tian and Yu (2017) explored the effects of ATL-2 on the gastric carcinoma cell lines HGC-27 and AGS and demonstrated that ATL-2 inhibits cell proliferation and migration by inducing apoptosis through the mitochondrial pathway, specifically by increasing the Bax/Bcl-2 ratio and suppressing the Akt/ERK signaling pathways. Collectively, these studies underscore the potential of AMK extract and its components to serve as therapeutic agents for GI cancers. The cytotoxic and apoptotic effects of AMK in various cancer models were achieved through immune modulation, apoptosis induction, and pathway-specific inhibition. These findings suggest its potential as a therapeutic option for GI cancer treatment.

Several studies have investigated the effects of AMK in various GI dysfunction models. For instance, Song et al. (2014), Song et al. (2015), Song et al. (2017), and Ren et al. (2021b) examined the effects of AMK extracts and their compounds on the migration and mucosal repair of intestinal epithelial cells, emphasizing their potential for the treatment of GI injuries. AMK extract accelerates the migration of intestinal epithelial cell during early mucosal repair by modulating the membrane potential and cytosolic Ca^2+^ levels in a polyamine-dependent manner, particularly by upregulating Kv1.1 channel mRNA and protein expression. The follow-up study conducted by Song et al. (2015) further revealed that the administration of AMK extract increased the expression of Rho mRNA and non-muscle myosin 2 protein and promoted stress fiber formation. These processes are all mediated by polyamine signaling, underscoring the role of polyamines in facilitating cell migration into the wound area. Song et al. (2017) also investigated the effect of ATL-1 on rat-derived IEC-6 cells and reported that ATL-1 enhanced epithelial cell migration and proliferation by increasing the polyamine and intracellular calcium levels, which are critical for mucosal healing, thereby supporting the use of ATL-1 as a potential agent for GI repair. Ren et al. (2021b) compared four active ingredients of AMK and identified ATL-3 as the most effective compound for promoting intestinal epithelial repair. ATL-3 exerts significant effects on cell migration, proliferation, and anti-inflammatory responses by increasing the intracellular calcium levels and upregulating the key proteins and cytokines associated with cellular repair and inflammation reduction. Thus, AMK and its components are promising candidates for therapeutic applications in gastrointestinal mucosal injuries, as they can potentially enhance epithelial healing by increasing polyamine and intracellular calcium levels.

Xu et al. (2023) examined the cellular mechanisms of ATL-1, a major bioactive component of AMK, in a model replicating the oxidative stress-induced colonic mucosal epithelial cell dysfunction observed in IBS. ATL-1 effectively alleviated oxidative stress-induced dysfunction by improving the cell viability and reducing apoptosis. Treatment with ATL-1 suppresses the expression of miR-34a-5p induced by oxidative stress, which directly targets lactate dehydrogenase A (LDHA), a key enzyme in glucose metabolism. ATL-1 restored glucose metabolism through the modulation of the miR-34a-5p pathway, thereby reducing colonic epithelial cell injury and maintaining metabolic homeostasis.

Xie et al. (2013) examined the effects of AMK on enteric mucosal immunity in a mice model injected with a foot-and-mouth disease vaccine. AMK polysaccharides enhanced the serum-specific IgG response and improved gut mucosal immunity as evidenced by the increase in the total SIgA concentration, elevated mRNA expression of TGF-β, IL-6, and TNF-α, increase in the IgA+ cell area, and a higher number of intraepithelial lymphocytes in the duodenum. Thus, AMK enhances the local mucosal immune response.

In summary, AMK and its active compounds demonstrate therapeutic potential in various GI disorders, including colitis (IBD), diarrhea, constipation, IBS, gastritis, gastric ulcers, and GI cancers. AMK restores intestinal function through its anti-inflammatory and antioxidant effects in patients with inflammatory and functional GI disorders. Furthermore, it promotes mucosal healing, and exerts a positive influence on the gut microbiota, particularly through the modulation of tryptophan metabolism, a pathway implicated in IBD and IBS. AMK exerts protective effects through the NF-κB-related pathways in gastric disorders such as gastritis and gastric ulcers, thereby reducing inflammation and oxidative stress while enhancing gastric mucosal defenses. Extensive studies using GI cancer models have revealed that AMK compounds, especially ATL-1 and ATL-3, inhibit tumor growth through the TLR4/MyD88 pathways and mitochondrial signaling. In addition, they enhance immune responses and synergize with chemotherapy by targeting cancer cell apoptosis and proliferation. *In vitro* studies underscored the role of AMK in epithelial repair and barrier function enhancement, suggesting its efficacy in restoring GI mucosal integrity. Thus, AMK holds promise as a multifaceted therapeutic agent that supports GI health, modulates inflammation, enhances epithelial repair, and provides anticancer benefits. It is particularly valuable for the treatment of GI cancers and broader gastrointestinal diseases.

#### 3.3.3 Alisma canaliculatum A.Braun & C.D.Bouche

The two studies on ‘*A. canaliculatum A. Braun* *& C.D.Bouche* (AC) included in this review comprised one *in vivo* study and one *in vitro* study (Table 5). Kim et al. (2018) reported the anti-inflammatory effects of the AC extract in gastritis and colitis models in *in vitro* models. The anti-inflammatory mechanism was mediated through the suppression of NF-κB/AP-1 signaling pathways. The AC extract inhibited the activation of NF-κB and AP-1, which play crucial roles in inflammatory responses, at the molecular level. The extract also inhibited the upstream signaling enzymes associated with NF-κB (Src and Syk) and AP-1 (TAK1 and MMK4/7). These findings indicate that the suppression of these enzymes mediates the anti-inflammatory potential of AC extract by targeting the NF-κB/AP-1 pathways.

**TABLE 5:** Studies of *Alisma canaliculatum A.Braun & C.D.Bouche* on gastrointestinal function.

Study	Model	Species or cell	Inducer	Dose/Route/Regimen	Results
*in vivo*					
Kim et al. (2018)	Gastritis	Male ICR mice (15–20 g)	Oral administration of 400 μl of 60% ethanol in 150 mM HCl 30 min after testing	70% ethanol AC extract 200 mg/kg p.o. (two times for 3 days)	AC extract • Ameliorated inflammatory symptoms associated with gastritis and colitis. • Reduced the levels of phospho-AKT and phospho-IκBα • Suppressed the increase in the TAK1 and MAPKK levels
	Colitis		Oral administration of 3% DSS	70% ethanol AC extract 200 mg/kg p.o. for 7 days	
*in vitro*					
Kim et al. (2018)		RAW264.7 cell	LPS 1 μg/mL for 24h	70% ethanol AC extract 0–400 μg/mL for 30 min before challenge	AC extract • Suppressed NO and PGE2 production • Reduced expression of iNOS, TNF-α, and COX-2 • Decreased NF-κB-mediated luciferase and CREB activity • Inhibited nuclear translocation of NF-κB subunit p65 and AP-1 subunit c-Jun • Suppressed the phosphorylated levels of NF-κB regulatory proteins (phospho-IκBα, IKK, Scr, Syk, and p85/PI3K) and AP-1 related MAPKs (phospho-JNK, p38, and ERK), as well as MAPKKs (MKK4/7, MKK3/6, and MEK1/2) The NK-κB/AP-1-targeted anti-inflammatory potential of AC extract is mediated by suppression of Src/Syk, complex formation between TAK1 and its substrate proteins MKK4/7
		HEK293 cell			
Kwon et al. (2021)	—	AGS gastric cancer cell	—	AC methanol extract 20 μL	AC extract • Inhibited AGS cell growth • Increased proportion of sub-G1 phase cells and mitochondrial membrane depolarization • Decreased Bcl-2 levels and survivin expression, with increasing Bax levels • Activated caspase-3 and caspase-9, leading to PARP cleavage • Elevated phosphorylation of p38 and ROS levels • AC extract-induced apoptosis is dependent on caspase and mitochondrial pathways in AGS cells.

ICR: institute of cancer research, min: minute, p. o.: per oral, LPS: lipopolysaccharide, h: hour, min: minute, NO: nitric oxide, PGE2: Prostaglandin E2, iNOS: inducible NO, synthase, TNF-α: Tumor necrosis factor-α, COX-2: Cyclooxygenase-2, NF-κB: Nuclear factor-κB, CREB: cAMP, response element binding protein, AP-1: Activator protein-1, IκBα: Inhibitor of κBα, Syk: spleen tyrosine kinase, PI3K: Phosphoinositide 3 kinase, MAPK: mitogen activated protein kinase, JNK: c-Jun N-terminal kinase, ERK: extracellular signal regulated kinase, MAPKK: mitogen activated protein kinase, TAK1: Transforming growth factor beta-activated kinase 1, MEK: ERK, kinase, PARP: Poly ADP-ribose polymerase, ROS: reactive oxygen species.

Kwon et al. (2021) reported the anti-cancer effects of the AC extract on AGS gastric cancer cells. The extract suppressed the proliferation of AGS cell and induced apoptosis. Furthermore, it activated caspase-3 and caspase-9 and increased the phosphorylation of p38, indicating that AC extract-induced apoptosis involves the mitochondrial pathways and MAPK cascades.

Thus, AC exhibited anti-inflammatory and anti-cancer effects in the GI disease model, indicating its potential as a therapeutic agent.

## 4 Discussion

Nine *in vivo*, one *in vitro*, and four human studies were analyzed in the present study to understand the research trends in BGT. The findings of the present study confirm that BGT and its modified forms exhibit anti-inflammatory effects, promote recovery of the GI mucosa, and regulate GI motility. BGT has been widely used for the treatment of diarrhea in Korea. Nevertheless, studies on the effects of BGT remain limited despite its effectiveness, making it difficult to understand the effects and mechanisms of action despite its efficacy. Therefore, the present study summarized all available studies on BGT and analyzed the effects of its key components to clarify its therapeutic potential and mechanisms.

### 4.1 Review of the main results

Studies on the efficacy of BGT have primarily focused on IBD, including UC and Crohn’s disease (CD), which are characterized by chronic relapsing intestinal inflammation (Zhang and Li, 2014). The pathogenesis of IBD involves the activation of T cells, monocytes, and macrophages (Fujino et al., 2003). TNF-α, IL-1β, IL-6, IFN-γ, IL-10, IL-12, and IL-17 are pro-inflammatory cytokines that exacerbate mucosal inflammation in IBD. Recent studies on the treatment strategies for IBD have focused on reducing inflammation through the regulation of pro-inflammatory cytokines (Abraham et al., 2017). The present study revealed that BGT exerts an anti-inflammatory effect by reducing the levels of cytokines, especially those of TNF-α, IL-1β, and IL-17, which is consistent with these findings.

TNF-α plays a key role in the development of several chronic inflammatory disorders, including IBD. Notably, elevated TNF-α levels have been detected in the serum of patients with UC and CD. Biologic therapies targeting TNF-α administered intravenously or subcutaneously have transformed the treatment of IBD and emerged as the most effective agents for achieving and maintaining disease remission (Souza et al., 2023). Infliximab, an anti-TNF-α medication, has achieved and maintained remission in patients with moderate-to-severe IBD (Hanauer et al., 2002).

IL-1β, an agonistic ligand of the IL-1 family, is involved in the innate immune response. Furthermore, it serves as a key mediator of inflammation in various human disorders, including IBD (Garlanda et al., 2013). It recruits and activates immune cells within the gut mucosa, thereby promoting pro-inflammatory responses (Aggeletopoulou et al., 2024). Elevated IL-1β expression has also been observed in patients with IBD (McAlindon et al., 1998), and its levels have been positively correlated with the severity of mucosal inflammation (Al-Sadi et al., 2008). IL-1β also influences downstream T-cell responses, such as the activation of Th17 cells, which produce IL-17 (Aggeletopoulou et al., 2024).

IL-17 induces various inflammatory mediators, particularly those involved in the proliferation, maturation, and recruitment of neutrophils (Witowski et al., 2004). Notably, IL-17 also stimulates the production of IL-1β and TNF-α in macrophages, which amplify the inflammatory response. Elevated IL-17 levels have also been detected in the colorectal tissue and serum of patients with IBD (Fujino et al., 2003).

BGT has demonstrated anti-carcinogenic effects in various diarrheal models (Joun et al., 1994; Ro et al., 2006). Diarrhea, defined as the excretion of liquid-like stool owing to abnormally accelerated peristalsis in the small and large intestines, is attributed to impaired digestion. Thus, accelerated intestinal motility is a key factor involved in the development of diarrhea (Yamamoto et al., 1993). BGT suppresses transport in the small and large intestines. Furthermore, it inhibits the contraction of ileal smooth muscle to exert an anti-cathartic effect. However, the results of the studies vary. Four clinical studies indicated that BGT alleviates the symptoms of diarrhea in patients with IBS and IBD. Notably, BGT depolarized and reduced the pacemaker potential frequency in ICCs to generate “slow waves,” i.e., regular contractions of the GI smooth muscles, in an *in vitro* study (Choi et al., 2023). GI tract motor dysfunction is a leading cause of GERD and FD in Korea (Noh et al., 2010; Sinn et al., 2010). These findings indicate the utility of BGT in regulating GI motility. While the studies suggest that BGT has the potential to alleviate symptoms of GI motility disorders, such as IBS and FD, the limitations of these studies highlight the need for further research. The clinical research was constrained by small sample sizes, a lack of objective assessment tools, and the absence of RCTs. Therefore, well-designed clinical studies are essential to confirm these promising findings.

The effect of BGT on the intestinal microflora was evaluated in the study conducted by Kim et al. (1993). The administration of BGT increased the count of lactic acid bacteria in the gut and modulated β-glucuronidase activation in the antibiotic-pre-treated group. The levels of β-glucuronidase, a lysosomal enzyme involved in the breakdown of β-D-glucuronides, are elevated in necrotic areas and body fluids of patients with cancer (Awolade et al., 2020). These findings indicate that BGT may act as a prebiotic and aid in maintaining intestinal homeostasis.

Some studies have examined the anti-ulcer effects of BGT using peptic ulcer models induced by ethanol, indomethacin, or pylorus ligation (Nam et al., 1989; Joun et al., 1994). Notably, BGT inhibited ulcer formation and reduced gastric acid secretion and acidity in these studies. These anti-ulcer effects may be attributed to the suppression of gastric juice secretion or the reduction of gastric acidity.

Despite the emergence of these promising findings, the limited number of studies has led to the requirement to conduct further studies exploring individual herbs present in BGT to explore their effects and mechanisms. mBGT, a new, simple, and effective formulation that includes only *L. japonica*, *A. macrocephala*, and *A. orientalis,* the most potent herbs from BGT, was developed in recent years (Kim, 2017; Kim et al., 2021). The key herbs present in mBGT were analyzed in the present study.

The flowers of LJT, commonly known as *Jin Yin Hua* (金銀花), *Ren Dong* (忍冬), or Japanese honeysuckle, are widely used in traditional Chinese medicine to treat exopathogenic wind-heat, epidemic febrile diseases, sores, carbuncles, and infectious diseases (Shang et al., 2011). Numerous studies have reported that these therapeutic effects of LJT are similar to those of BGT. Studies on colitis have demonstrated the anti-inflammatory effects of LJT and its underlying mechanisms of action. Analysis of the cytokine levels and T cell populations has provided insights into the modulation of the Th1/Th17 pathway by LJT through the inhibition of the NLRP3 inflammasome. The NLRP3 inflammasome, which is present in the mucosal macrophages and dendritic cells, plays a crucial role in inflammation regulation by modulating the activation of pro-inflammatory cytokines such as IL-1β and IL-18. These cytokines drive gut inflammation and tissue damage. Furthermore, they promote the differentiation of naïve T cells into Th17 cells, which contribute to the development and persistence of inflammation (Coccia et al., 2012; Prossomariti et al., 2018). LJT increases the secretion of mucosal protective factors; reduces oxidative stress; and downregulates NF-κB expression, a key factor in mucosal inflammation, thereby protecting the gastric mucosa (Bang et al., 2019). LJT extract enhances the abundance of probiotics, reduces pathogenic bacteria, and promotes the production of gut microbiota-derived metabolites such as SCFAs. FMT further validated these effects. Previous studies have explored the effects of LJT on GI motility. LJT improves gastric emptying and GI transit by enhancing the contraction of circular muscles in the antrum, esophageal smooth muscle cells, and lower esophageal sphincter muscle. Thus, the gastroprokinetic effects of LJT are mediated through the cholinergic and serotonergic pathways, as supported by neurotransmitter analysis and electric stimulation. LJT extract alleviated symptoms of FD and reduced the levels of 8-OhdG, a biomarker of oxidative DNA damage, in a clinical study. These findings suggest that the gastroprokinetic effects of LJT, consequently those of BGT, could aid in the treatment of GI motility disorders, including FD.

*Atractylodes macrocephala Koidz.* (AMK), also known as “Baizhu (白朮)” in traditional Chinese medicine, has been used to treat various conditions, including spleen hypofunction, loss of appetite, abdominal distension, diarrhea, dizziness, and heart palpitation, for thousands of years (Shu et al., 2017). AMK rhizome exhibits various biological activities, such as enhanced GI function and anti-tumor, anti-inflammatory, anti-aging, anti-oxidant, anti-osteoporotic, anti-bacterial, and neuroprotective effects (Zhu et al., 2018). The studies included in this review provide evidence for these therapeutic properties. The significant anti-inflammatory effects exerted by AMK in patients with various GI disorders such as colitis, gastritis, and gastric ulcer can be largely attributed to its ability to regulate key cytokine pathways. AMK inhibits pro-inflammatory cytokines such as TNF-α, IL-6, and IL-1β. In addition, it reduces immune cell infiltration in the inflamed tissues, thereby decreasing local inflammation. NF-κB and IL-6/STAT3, which play pivotal roles in the chronic inflammation of the GI tract, suppress key pathways to mediate these anti-inflammatory actions. AMK components such as ATL-1 and ATL-3 modulate oxidative stress and cytokine production by downregulating these pathways, thereby preventing further tissue damage, and promoting the recovery of the inflamed GI tissues. AMK also has a positive effect on the composition of the gut microbiota, which plays a vital role in host physiology. This effect is mediated by metabolites produced by microbes or derived from the transformation of host or environmental molecules (Devlin et al., 2016). AMK treatment increased the abundance of beneficial gut microbiota and the production of SCFAs in models of colitis and other GI disorders. Specific studies have highlighted the effect of AMK on the metabolism of tryptophan, which produces metabolites linked to anti-inflammatory responses and mucosal healing. Alterations in the metabolism of tryptophan play an active role in the pathogenesis of IBD. Dysfunction in the production of serotonin has been linked to the development of IBS (Agus et al., 2018). Cheng et al. (2023) proposed that AMK may act as a prebiotic for UC by modulating the gut microbiota metabolism, particularly the metabolism of tryptophan. Hu et al. (2023), reported that AMK significantly increased the levels of AhR ligands in a DSS-induced colitis model, indicating that AMK ameliorates IBD by regulating the metabolism of tryptophan. Similarly, AMK normalized the levels of tryptophan metabolites (such as 5-HT, indole, and kynurenic acid) and the expression of related enzymes (such as tryptophan hydroxylase, monoamine oxidase, and IDO) in constipation models (Yang et al., 2023; Qin et al., 2024). AMK modulates microbial metabolites and pathways, such as tryptophan and bile acid metabolism, to support a balanced microbiome, which is essential for managing conditions such as colitis and constipation. This finding further underscores its therapeutic potential. The ability of AMK to enhance the integrity of the intestinal barrier is a key mechanism that supports GI health. AMK polysaccharides increase the expression of tight junction proteins, such as ZO-1, Occludin, and Claudin-1, to strengthen the intestinal epithelial barrier in patients with conditions such as colitis and diarrhea. Barrier reinforcement reduces gut permeability and protects the gut from harmful pathogens and toxins. AMK also increases the production of mucins, such as MUC2, which coat the gut lining, thereby exerting protective effects and supporting epithelial repair. The ability of AMK to maintain the intestinal barrier aids in symptomatic relief. Moreover, it supports long-term GI health by mitigating gut dysfunction and inflammation. AMK and its active compounds, ATL-1 and ATL-3, have demonstrated promising anti-cancer effects in GI cancer models by inhibiting tumor growth and proliferation through the induction of apoptosis and modulation of the immune system. AMK activates TLR4, leading to enhanced immune cell activity against tumors through the MyD88 pathway. Moreover, the effect of AMK on signaling pathways such as Wnt/β-catenin, TLR4/MyD88, and mitochondrial pathways suppress cancer cell proliferation, increase cancer cell apoptosis, and improve responses to chemotherapy agents. Thus, AMK exhibits significant potential as a complementary therapeutic agent for GI cancers owing to its ability to target cancer cell growth and enhance immune responses. In summary, the therapeutic effects of AMK in GI disorders stem from multifaceted mechanisms, including anti-inflammatory, microbiota-modulatory, barrier-enhancing, and anti-tumor activities. These properties suggest that AMK is a promising agent for the management of functional and inflammatory GI disorders, as well as GI cancers, thereby providing comprehensive support for GI health and stability.

Rhizoma Alismatis (澤瀉), the dried rhizome of the plant *A. canaliculatum A. Braun* *& C.D.Bouche* (AC) from the Alismataceae family, is commonly used in traditional Chinese medicine to treat conditions such as dysuria, edema, nephropathy, hyperlipidemia, diabetes, and inflammation (Tian et al., 2014). Studies on AC remain limited; however, it exhibits anti-inflammatory effects in gastritis and colitis models through NF-κB-related mechanisms and anti-tumor effects in gastric cancer models through mitochondrial pathways. These therapeutic effects were similar to those observed for AMK, contributing to our understanding of the potential benefits of BGT.

Quality assessment of the data was conducted in accordance with the Animal Research: Reporting of *In Vivo* Experiments (ARRIVE) 2.0 guidelines (Percie du Sert et al., 2020) using a 21-item checklist. A total of 43 articles that met the eligibility criteria were included in the study. The ARRIVE scores ranged from 0 to 100, based on whether the specific items were reported. The bar graph displays all the scores assigned to each of the 43 studies, from the lowest (33%) to the highest (86%) (Supplementary Figure S4). The mean checklist score was 69%, which falls into the medium adherence category (50%–79%). The items that received the highest scores included the number of animals used, the size of control and experimental groups, and the definition of experimental outcomes. Conversely, the least frequently reported items were related to housing and husbandry, animal care and monitoring, protocol registration, and generalizability (Supplementary Figure S5). Our study found that the quality of reporting for key items that influence the interpretation of preclinical studies was generally poor. Notably, details crucial for ensuring the reproducibility of preclinical studies, such as animal housing, husbandry, and anesthetics, were often underreported, potentially impacting study results. This highlights a limitation in the overall quality of research adhering to the ARRIVE guidelines and suggests a broader shortfall in ethical considerations within current animal research. Therefore, it is essential for future *in vivo* studies to not only comprehensively implement the ARRIVE guidelines but also to address these ethical gaps. This will facilitate the effective translation of preclinical research findings into clinical therapies while ensuring adherence to ethical principles and promoting the conduct of high-quality animal research.

### 4.2 Strengths and limitations of the study

This study had certain limitations that must be addressed. First, studies on BGT are limited. The lack of high-quality evidence, such as those obtained through RCTs or SRs, and the limited number of experimental studies made gaining a precise understanding of the efficacy and mechanisms of BGT challenging. Additionally, a significant limitation arises from the relying on preclinical study outcomes as the sole predictors of clinical efficacy. While preclinical models provide valuable insights into initial safety and mechanisms, they often fail to replicate the complex interactions present in human physiology. This discrepancy underscores the need for caution when extrapolating these findings to clinical settings. Therefore, it is crucial to advance toward meticulously designed clinical trials that build upon preclinical research. Such trials should incorporate robust methodologies, diverse patient populations, and comprehensive endpoints to adequately evaluate the therapeutic potential and safety profile of interventions like BGT. Only through this rigorous translational approach can the true clinical benefits be ascertained, thereby enhancing the reliability and applicability of the research outcomes. Second, confirming the interactions among the three primary herbal components analyzed in this review was difficult. Herbal medicines are characterized by the synergistic effects arising from a combination of multiple herbs. The effects of the individual herbal components were evaluated in the present review; however, whether the same mechanisms would be retained when the three herbs or additional components are combined remains uncertain. Further studies, including experimental studies and large-scale clinical studies involving diverse populations, must be conducted in this regard to elucidate the mechanisms of action of BGT. In conclusion, addressing the limitations identified in this study is crucial to substantiate the clinical efficacy of BGT and its role within the broader therapeutic spectrum. Achieving this will require well-structured preclinical and clinical studies designed to elucidate the complex interactions and mechanisms of BGT. Such research should include diverse populations to comprehensively evaluate the therapeutic potential of BGT.

Despite these limitations, the present study also has several strengths. Our primary objective was to provide a comprehensive synthesis of current knowledge regarding BGT and its effects on the GI tract. By summarizing existing studies, we aimed to identify gaps in the literature and suggest areas for future research, thereby offering value to both practitioners and researchers. To the best of our knowledge, this is the first scoping review to comprehensively synthesize the effects and mechanisms of BGT on GI function. The findings of this scoping review extend the therapeutic potential of BGT, which has primarily been studied in the context of colitis, to a broader range of GI symptoms. Additionally, by analyzing the effects and mechanisms of the main herbal components of BGT, we could infer the mechanisms and efficacy of BGT, which is widely used in clinical settings. The study attempted to assess the quality of *in vivo* studies using the ARRIVE Tool. This review uniquely integrates traditional and modern pharmacological insights, bridging the historical uses of BGT with contemporary scientific understanding. By focusing on the key components of BGT, our study highlights the synergistic effects of its herbal constituents, offering novel perspectives that contribute to the existing body of knowledge and underscore the therapeutic potential of this traditional formulation. Furthermore, this review visualized and presented the diverse mechanisms identified in the studies using diagrams, thereby enhancing the clarity and understanding of the findings.

## 5 Conclusion

**FIGURE 2:**
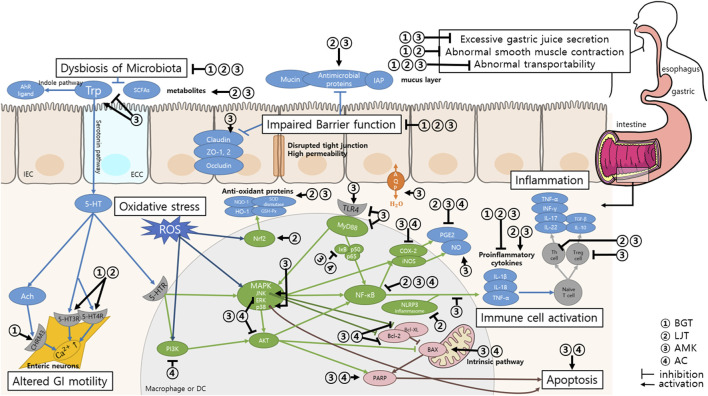
Summary of the mechanisms of action of *Bojanggunbi-tang* and its composing herbs on GI function. Changes in gut microbiota lead to reduced production of SCFAs and impaired Trp metabolism, contributing to disruptions in the serotonin pathway and intestinal barrier function. BGT and its components regulate this imbalance by promoting the growth of beneficial microbiota and modulating Trp metabolism. BGT and its components can regulate GI motility: LJT through the serotonergic pathways and BGT through the cholinergic and serotonergic pathways. Dysbiosis and oxidative stress impair intestinal barrier function, leading to decreased levels of tight junction proteins and increased gut permeability, which exacerbates inflammation. BGT and its components strengthen the intestinal barrier by promoting mucosal recovery, increasing the number of goblet cells, and regulating tight junction proteins. BGT reduces inflammation by modulating pro-inflammatory cytokines (TNF-α, IL-1β, and IL-17) and inhibiting key inflammatory pathways, such as NF-κB, TLR4, and the NLRP3 inflammasome. LJT and AMK regulate immune responses by controlling the Th1/Th17 pathways to achieve a balance between pro-inflammatory and anti-inflammatory cytokines. BGT components also exhibit antioxidant properties that enhance the activity of proteins such as Nrf2 and inhibit oxidative stress pathways (MAPK and NF-κB), thereby contributing to reduced ROS and apoptosis. Intrinsic apoptosis pathway, triggered by inflammation and oxidative stress, leads to cellular damage in the GI mucosa. BGT and its components, particularly AMK, inhibit mitochondrial pathways (BAX/Bcl-2), preventing excessive apoptosis and promoting tissue recovery. Abbreviations: BGT, Bojanggunbi-tang; LJT, *Lonicera japonica* Thunb.; AMK, Atractylodes macrocephala Koidz.; AC, Alisma canaliculatum A.Braun & C.D.Bouche; AhR, Aryl hydrocarbon receptor; Trp, Tryptophan; SCFAs, Short-chain fatty acids; IAP:, IEC, Intestinal epithelial cell; ECC, Enterochromaffin cell; ZO, Zonula occludens; AQP, Aquaporin; 5-HT, 5-hydroxytryptamine; Ach, Acetylcholine; 5-HTR, 5-HT receptor; CHRM2, Cholinergic receptor muscarinic 2; ROS, Reactive oxygen species; NQO-1, NAD(P)H: Quinone oxidoreductase-1; SOD, Superoxide dismutase; HO-1, Heme oxygenase-1; GSH-Px, Glutathione peroxidase; Nrf2, Nuclear factor erythroid-2-related factor 2; MAPK, Mitogen-activated protein kinase; JNK, c-Jun N-terminal kinase; ERK, extracellular signal-regulated kinase; p38, p38 mitogen-activated protein kinases; AKT, Serine/Threonine protein kinase; TLR4, Toll-like receptor 4; MyD88, Myeloid differentiation primary response gene 88; IκB, Inhibitor of NF-κB; NF-κB, Nuclear transcription factor-κB; COX-2, Cyclolxygenase-2; iNOS, inducible NO synthase; PGE2, Prostaglandin E2; NO, Nitric oxide; NLRP3, nucleotide-binding domain-like receptors family pyrin domain containing 3; IL, Interleukin; INF, Interferon; TGF, Transforming growth factor; Th cell, helper T cell; Treg cell, regulatory T cell; Bcl-2, B-cell lymphoma-2; Bcl-xL, B-cell lymphoma-extra-large; BAX, Bcl-2-associated X protein; PARP, Poly ADP ribose polymerase.

BGT and its key herbal components, *L. japonica Thunb.*, *A. macrocephala Koidz.*, and *A. canaliculatum A. Braun* *& C.D.Bouche,* are multifaceted therapeutic agents for the management of GI disorders. The anti-inflammatory, microbiota-modulatory, intestinal barrier-enhancing, and anti-tumor effects of BGT exhibit promise for the management of functional and inflammatory GI conditions, including diarrhea, IBD, and GI cancers. BGT and its active components can address multiple facets of GI health and disease by modulating key inflammatory cytokines, supporting microbiome balance, reinforcing the intestinal barrier, and targeting cancer-related pathways (Figure 2). These findings underscore the therapeutic versatility of BGT, indicating its potential for broader clinical applications as an effective natural intervention for GI health and stability. Nevertheless, further studies, particularly clinical studies, must be conducted in the future to deepen our understanding of the underlying mechanisms and optimize the clinical use of BGT.

## Data Availability

The original contributions presented in the study are included in the article/Supplementary Material, further inquiries can be directed to the corresponding author.
