# Supplementary material for: Effect of Bojanggunbi-tang and its primary constituent herbs on the gastrointestinal tract: a scoping review
Source: Front Pharmacol. 2025 Mar 12;16:1543194. doi: 10.3389/fphar.2025.1543194 (PMC11938064; doi:10.3389/fphar.2025.1543194)
Supplement: Supplementary file 1 [file DataSheet1.docx]

Supplementary Material

**Table S1. Search Strategy for *Bojanggunbi-tang***

Medline via PubMed

| Searches | |
| --- | --- |
| #1 | "Bojanggunbi-tang"[All Fields] OR "Bojanggunbitang"[All Fields] |
| #2 | "gastrointestinal"[All Fields] OR "gastrointestinally"[All Fields] OR "gastrointestine"[All Fields] OR "gastr*"[All Fields] OR "intestin*"[All Fields] OR ("colon"[MeSH Terms] OR "colon"[All Fields] OR "colonic"[All Fields] OR "colons"[All Fields] OR "colon s"[All Fields] OR "colonal"[All Fields] OR "colonically"[All Fields] OR "colonitis"[All Fields]) OR ("bowel s"[All Fields] OR "bowell"[All Fields] OR "intestines"[MeSH Terms] OR "intestines"[All Fields] OR "bowel"[All Fields] OR "bowels"[All Fields]) OR ("colitis"[MeSH Terms] OR "colitis"[All Fields] OR "colitides"[All Fields]) OR ("crohn disease"[MeSH Terms] OR ("crohn"[All Fields] AND "disease"[All Fields]) OR "crohn disease"[All Fields] OR "crohn s"[All Fields] OR "crohn"[All Fields] OR "crohns"[All Fields]) |
| #3 | #1 AND #2 |

CENTRAL

| Searches | |
| --- | --- |
| #1 | (Bojanggunbi-tang OR Bojanggunbitang OR Bojangkunbi-tang OR 補腸健脾湯):ti,ab,kw |
| #2 | (gastrointestinal OR gastr* OR intestin* OR colon OR bowel OR colitis OR crohn):ti,ab,kw |
| #3 | #1 AND #2 |

EMBASE

| Searches | |
| --- | --- |
| #1 | 'bojanggunbi tang' OR bojanggunbitang OR 'bojangkunbi tang' OR 補腸健脾湯 |
| #2 | ‘gastrointestinal’/exp OR gastrointestinal OR gastr OR intestine OR ‘colon’/exp OR colon OR ‘bowel’/exp OR bowel OR crohn |
| #3 | #1 AND #2 |

AMED

| Searches | |
| --- | --- |
| #1 | TX (Bojanggunbi-tang OR Bojanggunbitang OR Bojangkunbi-tang OR 補腸健脾湯) |
| #2 | TX (gastrointestinal OR gastr* OR intestin* OR colon OR bowel OR colitis OR crohn) |
| #3 | #1 AND #2 |

CNKI

| Searches |
| --- |
| 補腸健脾湯 OR Bojanggunbi-tang OR Bojanggunbitang OR Bojangkunbi-tang |

CiNii

| Searches |
| --- |
| 補腸健脾湯 OR Bojanggunbi-tang OR Bojanggunbitang OR Bojangkunbi-tang |

Kmbase

| Searches |
| --- |
| ((((보장건비탕\|total) OR (bojanggunbi-tang\|total)) OR (bojanggunbitang\|total)) OR (bojangkunbi-tang\|total)) OR (bojangkunbitang\|total) |

KISS

| Searches |
| --- |
| 전체 = "보장건비탕" or 전체 = "bojanggunbi-tang" or전체 = "bojanggunbitang" or전체 = "bojangkunbi-tang" or전체 = "bojangkunbitang" |

NDSL

| Searches |
| --- |
| 전체=보장건비탕 OR 전체=補腸健脾湯 OR 전체=bojanggunbi-tang OR 전체=bojanggunbitang OR 전체=bojangkunbi-tang OR 저자=bojangkunbitang |

OASIS

| Searches |
| --- |
| 보장건비탕 OR補腸健脾湯 OR bojanggunbi OR bojangkunbi |

**Table S2. Search Strategy for Herbs**

Medline via PubMed

| Searches | |
| --- | --- |
| #1 | "lonicera"[MeSH Terms] OR "lonicera"[All Fields] OR "lonicerae"[All Fields]) AND ("japonica"[All Fields] OR "japonicae"[All Fields] OR "japonicas"[All Fields] |
|  | "atractylodes"[MeSH Terms] OR "atractylodes"[All Fields] OR "atractylode"[All Fields]) AND ("macrocephala"[All Fields] OR "macrocephalae"[All Fields] |
|  | "alisma"[MeSH Terms] OR "alisma"[All Fields] |
| #2 | "gastrointestinal"[All Fields] OR "gastrointestinally"[All Fields] OR "gastrointestine"[All Fields] OR "gastr*"[All Fields] OR "intestin*"[All Fields] OR ("colon"[MeSH Terms] OR "colon"[All Fields] OR "colonic"[All Fields] OR "colons"[All Fields] OR "colon s"[All Fields] OR "colonal"[All Fields] OR "colonically"[All Fields] OR "colonitis"[All Fields]) OR ("bowel s"[All Fields] OR "bowell"[All Fields] OR "intestines"[MeSH Terms] OR "intestines"[All Fields] OR "bowel"[All Fields] OR "bowels"[All Fields]) OR ("colitis"[MeSH Terms] OR "colitis"[All Fields] OR "colitides"[All Fields]) OR ("crohn disease"[MeSH Terms] OR ("crohn"[All Fields] AND "disease"[All Fields]) OR "crohn disease"[All Fields] OR "crohn s"[All Fields] OR "crohn"[All Fields] OR "crohns"[All Fields]) |
| #3 | #1 AND #2 |

CENTRAL

| Searches | |
| --- | --- |
| #1 | (lonicera japonica):ti,ab,kw |
|  | (atractylodes macrocephala):ti,ab,kw |
|  | (alisma):ti,ab,kw |
| #2 | (gastrointestinal OR gastr* OR intestin* OR colon OR bowel OR colitis OR crohn):ti,ab,kw |
| #3 | #1 AND #2 |

EMBASE

| Searches | |
| --- | --- |
| #1 | ‘lonicera japonica’/exp OR ‘lonicera japonica’ |
|  | atractylodes AND macrocephala:ta,ab,kw |
|  | 'alisma'/exp OR 'alisma' |
| #2 | ‘gastrointestinal’/exp OR gastrointestinal OR gastr OR intestine OR ‘colon’/exp OR colon OR ‘bowel’/exp OR bowel OR crohn |
| #3 | #1 AND #2 |

Web of science

| Searches | |
| --- | --- |
| #1 | lonicera japonica (All Fields) |
|  | atractylodes macrocephala (All Fields) |
|  | Alisma (All Fields) |
| #2 | gastrointestinal OR gastr* OR intestin* OR colon OR bowel OR colitis OR crohn (All Fields) |
| #3 | #1 AND #2 |

**Figure S1. PRISMA flow diagram of study selection for *Lonicera japonica Thunb.***

**
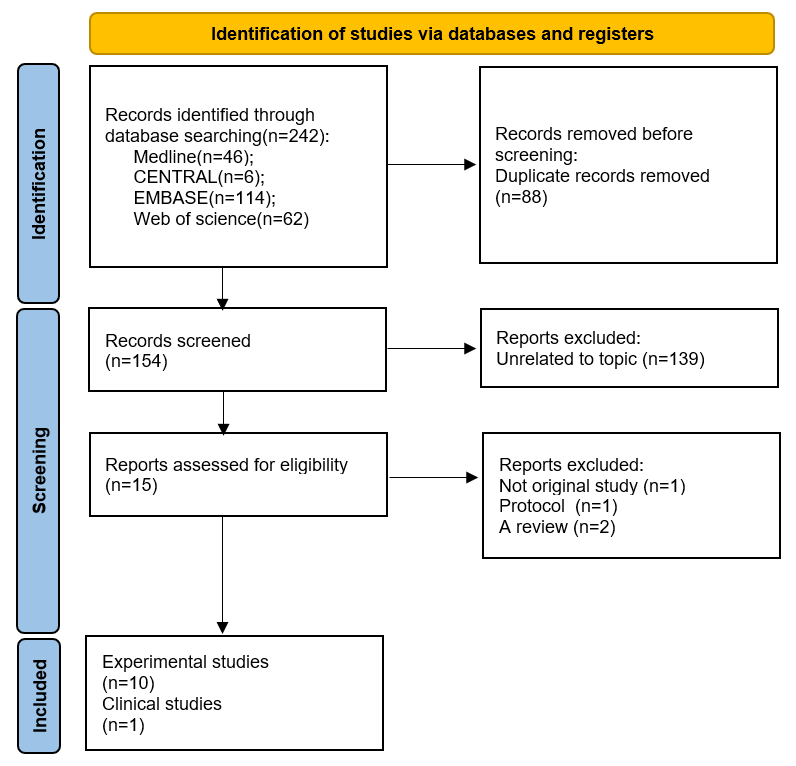
**

**Figure S2. PRISMA flow diagram of study selection for *Atractylodes macrocephala Kodiz.***

**
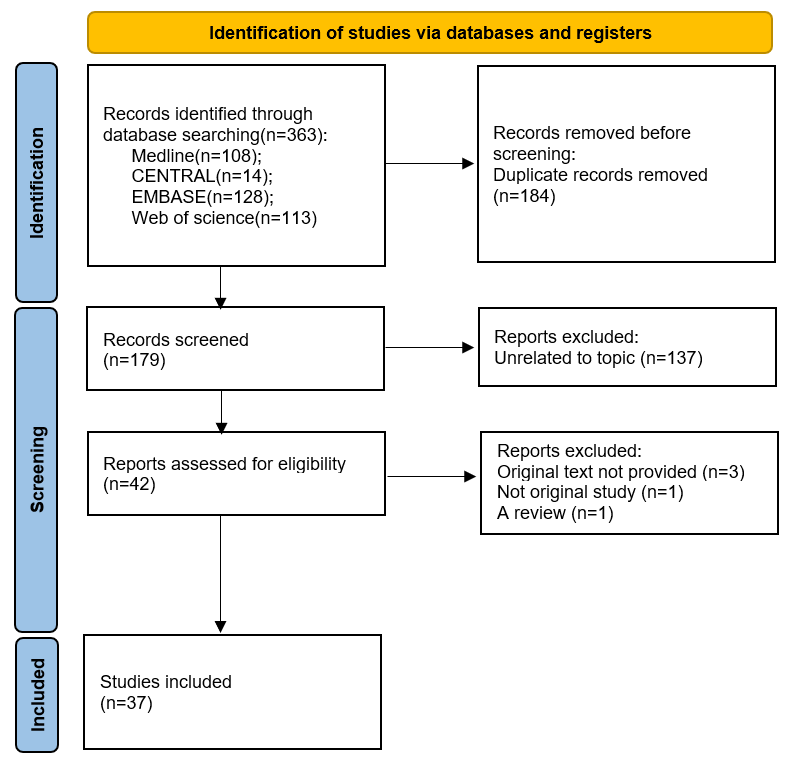
**

**Figure S3. PRISMA flow diagram of study selection for *Alisma canaliculatum A.Braun & C.D.Bouche***

**
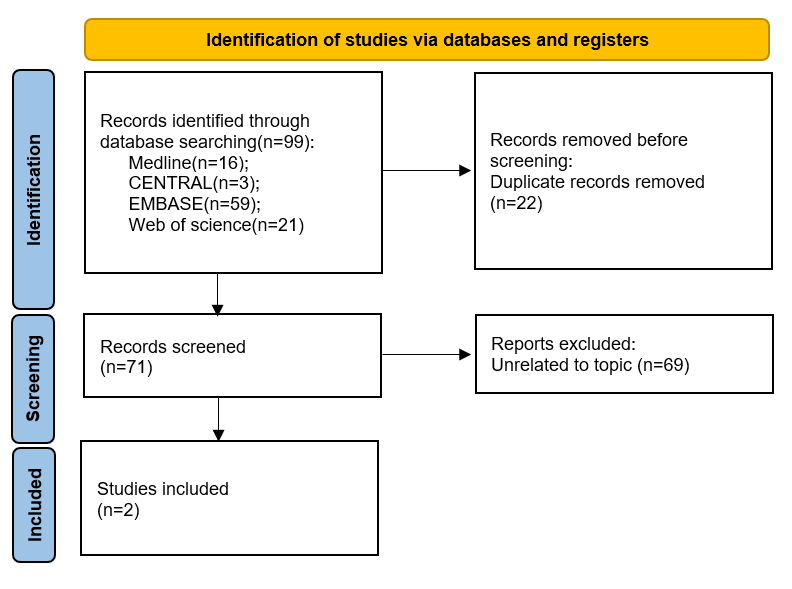
**

**Figure S4. Bar chart displaying the scores of the analyzed *in vivo* articles, representing the percentage adherence to the ARRIVE guidelines 2.0 on a scale from 0 to 100. Data with high degree of adherence (between 80 and 100%) are marked in blue, medium degree of adherence (between 50% and 80%) in yellow, and those with low degree of adherence (between 0 and 49%) in red.**

**< 50% 50-80% ≥ 80%**

**Figure S5. Bar chart showing the degree of adherence (in %) to the 21 items in the ARRIVE guidelines 2.0 checklist. The data were clustered to show the items where studies exhibited a high degree of adherence (between 80 and 100%, marked in blue), a medium degree of adherence (between 50 and 79%, marked in yellow), and a low degree of adherence (between 0 and 49%, in red). Each item is identified by its corresponding numbers and a label related to its content.**
